# CoMind R1: a time-resolved interferometric optical neuromonitoring system for pulsatile cerebral blood flow measurement at late times-of-flight

**DOI:** 10.1117/1.NPh.13.2.025002

**Published:** 2026-03-30

**Authors:** Veronika Parfentyeva, Anurag Behera, Stella Avtzi, Alexandra Tran-Van-Minh, Octave Etard, Suzanna Freer, Tanvi Tambe, Ali Mehmed, Artur Isufaj, Jan Goodrich, Youssef Ibrahim, Navjit Singh, Saeed Darabi, Pablo Villar Sanjurjo, Sujit Malde, Taimoor Ali, Yoojin Kim, Simone Sturniolo, Alexander Ruesch, Dominic William Hill, Amir Salehi Lashkajani, Yuqian Zhang, Patrick McCarthy, Xuesong Hu, Alexander Antrobus, Chloe Maine, Matt Thackrah, Matthew Taylor Valley, Benjamin Crutchley, Dawid Borycki, Tanja Dragojević, Claus Lindner, Robert James Cooper

**Affiliations:** a CoMind Technologies Ltd., London, United Kingdom; b University College London, Biomedical Optics Research Laboratory, Department of Medical Physics and Biomedical Engineering, London, United Kingdom

**Keywords:** interferometry, diffuse optics, pulsatile cerebral blood flow, time-of-flight, functional activation

## Abstract

**Significance:**

Continuous, noninvasive monitoring of cerebral blood flow (CBF) at the bedside is a critical unmet clinical need and a long-standing goal of biomedical optics. Despite extensive development, no optical measurement of CBF has achieved routine clinical use.

**Aim:**

We aim to demonstrate and validate CoMind R1, a time-resolved interferometric neuromonitoring system designed to noninvasively measure CBF at pulsatile rates.

**Approach:**

CoMind R1 integrates numerous advances, including a high-power, linearly swept 1064 nm laser, multimode collection, parallelized detection, and real-time processing. Performance is evaluated with homogeneous and dynamic bi-layer phantoms and *in vivo* studies of adults at rest and during visual stimulation.

**Results:**

CoMind R1 achieves an instrument response function width of 120 ps and exhibits no significant drift over 24 h. *In vivo* data from 25 adults show consistent pulsatile blood flow waveforms at times-of-flight averaging 1.2 ns, exceeding the prior art. Visual stimulation produces significant, time-of-flight-dependent hyperaemic responses, demonstrating brain sensitivity and selectivity.

**Conclusions:**

CoMind R1 consistently achieves time-of-flight resolved measurements of pulsatile blood flow at the late times-of-flight necessary to ensure a brain-dominated signal in the typical adult. CoMind R1 thus provides cerebral sensitivity and tissue specificity in a scalable, cost-effective architecture and advances optical monitoring of CBF toward clinical translation.

## 1 Introduction

Despite decades of development across numerous technological domains, no method for the continuous, noninvasive measurement of cerebral blood flow (CBF) at the bedside has achieved routine clinical adoption. Accurate, continuous monitoring of CBF would permit the early detection of perfusion deficits and enable timely intervention to reduce the risk of brain injury in a wide range of clinical settings, including critical care and surgery.^1^ It would also enable direct monitoring of cerebral autoregulatory state, which has the potential to guide patient-specific interventions to minimize secondary brain injury due to hypo- or hyperperfusion.^2^^,^^3^

Optical methods have long held potential for a noninvasive monitoring of the brain.^4^ The transmission of safe levels of light [particularly at near-infrared (NIR) wavelengths] through the biological tissues provides noninvasive access to a wide range of tissue characteristics. Most commonly, measurements of the relative optical absorbance of a sampled tissue at more than one wavelength are used to extract information about the concentration of the constituent absorbers in that sampled tissue.^5^ Such near-infrared spectroscopy (NIRS) approaches have been leveraged to noninvasively examine tissue hemodynamics, particularly tissue oxygenation, at the bedside since the 1990s,^6^^–^^8^ but their clinical utility has been limited by uncertainty in photon pathlength, the inability to separate absorption from scattering, and poor depth specificity. The need to overcome these limitations has driven the development of more advanced spectroscopic neuromonitoring methods, the most sophisticated being time-resolved or time-domain near infrared spectroscopy (TD-NIRS).^9^ These TD-NIRS methods employ photon counting techniques to determine the time-of-flight (ToF) of photons through the sampled tissue, providing a pathway to superior depth specificity when compared with traditional NIRS approaches.^10^

Alongside the ongoing efforts to measure brain tissue oxygen saturation, perhaps the second fundamental objective of optical neuromonitoring research over the last 30 years has been the noninvasive measurement of CBF.^11^^–^^13^ Over this period, a family of techniques have emerged that monitor blood flow by exploiting the temporal dynamics of the speckle patterns that are formed as coherent light travels diffusely through tissue. The rate of speckle intensity fluctuations is a function of the motion of optical scatterers within the sampled volume and is primarily driven by the motion of red blood cells in the microvasculature.^11^ Although the sensitivity of such measurements to other parameters (including optical scattering coefficient and hematocrit) has rendered the measurement of absolute blood flow difficult,^14^ the optical monitoring of relative changes in tissue blood flow is increasingly well established.^13^

The core technical challenge in the development of speckle-based methods of CBF monitoring is the necessity of resolving individual speckles and capturing their rapid intensity fluctuations, which requires a stable light source with a high temporal coherence. This is particularly challenging because to be sensitive to the brain, the collected light must have traversed many centimeters of tissue, resulting in speckles with low optical intensities. The sampling rate in the context of such measurements is also important because although pathological changes in CBF often unfold over minutes or hours, an effective clinical monitor must sample well above the frequency of cardiac pulsations as it has been shown that the CBF waveform itself encodes information of a high clinical value.^15^^–^^17^ Achieving an effective and clinically viable technology for the optical monitoring of CBF, therefore, requires a trade-off between the coupled parameters of sampling rate, signal-to-noise (SNR), brain sensitivity and selectivity, usability, and cost.

The most widely used method for the speckle-based measurement of cerebral blood flow dynamics is diffuse correlation spectroscopy (DCS). DCS quantifies blood flow by deriving a blood flow index (BFi) from the temporal autocorrelation of the speckle intensity time series captured from a single speckle. In standard DCS, the necessity of single-mode collection limits optical throughput, whereas a high-speed, high-sensitivity, and high-cost detector is required for every sampled speckle. As a result, the achievable SNR is directly tied to the number of detectors available, which is primarily constrained by cost.^11^

In an attempt to overcome the SNR limitations inherent to DCS, speckle contrast optical spectroscopy (SCOS) has emerged as a promising alternative.^18^ Unlike DCS, SCOS employs an imaging sensor to capture speckle patterns composed of thousands of speckles, and BFi is then extracted by analyzing temporal changes in speckle contrast. These imaging sensors typically have lower sensitivity per pixel and operate at frame rates below those of the high-speed, photon-counting detectors of DCS. However, the sheer number of pixels available enables SCOS methods to extract a BFi time series with an SNR that exceeds that of DCS under comparable conditions while also offering improved scalability and reduced system cost.^19^

As with all continuous-wave methods, achieving sufficient brain sensitivity with DCS and SCOS is predicated on achieving sufficiently large source-detector separations (SDSs). Increasing the SDS increases the average penetration depth of the collected photons and thus improves sensitivity to the brain tissue.^20^ However, for every additional centimeter of source–detector distance on the human head, there is an approximately 10-fold reduction in detected light intensity.^11^ This necessitates a careful trade-off between competing design priorities: maximizing brain sensitivity (via larger SDS) and maintaining adequate SNR at physiologically relevant sampling rates. In a typical multichannel DCS system, SDS values limited to 25 to 30 mm are necessary to maintain sufficient SNR when sampling at above pulsatile rates, whereas best-in-class SCOS systems have demonstrated SDS values up to 35 to 40 mm.^21^ These SDS ranges can provide reasonable brain sensitivity in the adult, exceeding that of comparable NIRS measurements. However, the resulting BFi time series will still contain a substantial but unknown contribution from superficial tissues, limiting their cerebral specificity and therefore utility in clinical settings.^22^

Despite the many significant successes of DCS and SCOS in laboratory and clinical research environments, neither technique has yet been translated successfully into a medical device. In addition to the challenges outlined above, usability is a critical consideration; both DCS and SCOS are highly sensitive to the ambient light,^11^ which can present a significant practical barrier to routine operation in clinical settings.

Combining the sensitivity to photon time-of-flight and capacity for the measurement of absolute optical properties offered by TD-NIRS methods with the sensitivity to speckle dynamics inherent to DCS has the potential to provide a multiparameter optical neuromonitoring technology that would be well positioned for clinical translation. Such an approach would enable the simultaneous measurement of ToF-resolved BFi and oxygenation, with the potential to separate blood flow signals originating in the superficial tissues from those originating in the brain, all from a single measurement.

The simultaneous measurement of the temporal point-spread function (TPSF) and ToF-resolved intensity autocorrelation functions was first undertaken by Yodh et al.^23^ who developed pulsed diffusing-wave spectroscopy where a ToF-gated autocorrelation function is measured using nonlinear optical methods. Further significant progress in this direction has been made in recent years through the development of time-domain DCS (TD-DCS).^10^^,^^24^ In TD-DCS, a pulsed long-coherence-length laser source is combined with single-mode fiber collection and single-photon counting detectors to measure the decay of the intensity field autocorrelation as a function of photon ToF. Such TD-DCS approaches have shown enhanced sensitivity to cortical activity relative to continuous wave DCS and have been used to successfully capture functional brain responses during cognitive and motor tasks and respiratory stimuli.^25^^,^^26^ However, achieving both pulsed operation and long coherence lengths presents conflicting requirements for laser source design, whereas the necessary detectors and timing electronics remain expensive and technically complex.^22^ The state-of-the-art TD-DCS devices rely on superconducting nanowire single-photon detectors,^25^^–^^27^ which are currently impractical for commercial translation. Furthermore, although such devices have successfully retrieved pulsatile BFi measurements at sampling rates of up to ∼30  Hz while incorporating late photon ToFs, they have typically exhibited a wide instrument response function (IRF)^28^ and/or required wide ToF gates spanning, e.g., 0.4 to 1 ns, to do so.^26^ Such wide ToF gates will capture a broad mix of superficial and deep photon paths, limiting brain specificity.

Using a different approach to integrating the benefits of both DCS and TD-NIRS, Borycki, Srinivasan et al. introduced a novel time-of-flight-resolved interferometric measurement method in 2016.^29^^–^^31^ This “interferometric NIRS” approach leveraged a frequency-swept 855 nm laser source in combination with a Mach–Zehnder interferometer, drawing on the principles used in swept-source optical coherence tomography.^32^ This enabled the extraction of ToF-resolved electric field autocorrelation functions. Mazumder et al.^33^ then extended this approach by employing a 1060 nm laser source to demonstrate that operation at longer wavelengths enhances the signal-to-noise ratio of ToF-resolved measurements of electric field autocorrelation and improves sensitivity to cerebral blood flow. By capturing both scattering dynamics and photon arrival times in a single modality, this method offers the potential to extract the full range of information provided by both DCS and TD-NIRS while simultaneously mitigating many of their respective technical constraints.

Such time-resolved interferometric measurements rely on single-mode collection and detection in a manner comparable to DCS, which limits optical throughput. However, the use of interferometric detection acts like an optical amplifier—the measured interference term is a product of the amplitudes of the electric fields of the reference and sample arms. The significantly attenuated field that emerges from the sampled tissue is therefore amplified prior to detection by the (much larger) field traversing the reference arm. This amplification makes it possible to obtain high-speed measurements of ToF-resolved speckle fluctuations using low-cost photodiode detectors, eliminating the need for single-photon detection. Interferometric detection, therefore, provides a path to highly parallelized systems that are not cost-prohibitive. Because photodiodes are a well established and ubiquitous technology, this detection approach is also readily scalable for manufacture as production would not be unduly dependent on a single, high-cost, hard-to-source detector component.

Direct measurement of the electric field autocorrelation (rather than the intensity autocorrelation) also removes the need to invoke the Siegert relationship, on which both DCS and TD-DCS depend,^11^ thereby avoiding uncertainty in the value of β and the potential for noise amplification.^29^ Last, interferometric detection intrinsically rejects external optical interference; the interference term is only significant for light that has precisely the same wavelength and a fixed phase relationship with the reference arm. Ambient light can contribute to the DC detected intensity, but this term can be effectively rejected via balanced detection.^34^ As a result, time-resolved interferometric measurements can be performed in a wide range of ambient lighting conditions without optical isolation, which is a critical advantage in the context of translation to clinical environments.

In recent years, several continuous wave (CW) methods that leverage some of the advantages of interferometric detection have also been introduced. These include interferometric diffusing wave spectroscopy (iDWS) and interferometric DCS (iDCS).^35^^,^^36^ Both permit the use of much larger SDS and thus increase brain sensitivity over prior CW methods. Extensions of these approaches have been developed that apply time-of-flight (ToF) gating to achieve depth-resolved measures of blood flow index, bridging the gap between CW and TD methods. For example, Zhao et al.^37^ demonstrated a coherence-gated iDWS system using a modulated laser source and showed improved suppression of superficial signal contributions. Robinson et al.^38^ reported a pulsed-laser coherence-gated iDCS instrument that combines ToF selectivity with single-shot correlation analysis, enabling pathlength-resolved flow measurements in scattering media. Because of their reliance on ToF gating, these systems cannot simultaneously obtain time-resolved BFi at multiple times-of-flight, but they do benefit from many of the advantages of interferometric detection, including field amplification and the direct measurement of the electric-field autocorrelation.

Time-resolved interferometric techniques that provide simultaneous access to both ToF-resolved BFi and the full TPSF have particularly high clinical potential. They provide a pathway to the measurement of multiple critical neurophysiological parameters, with high brain sensitivity and specificity in a single measurement and with a robust and scalable architecture. Although significant progress has been made in the development of such interferometric methods to date, all prior implementations have faced critical challenges—foremost among them is the difficulty of achieving sufficient SNR at the late photon ToFs that are essential for ensuring sufficient sensitivity to CBF.^39^ No interferometric NIRS system has yet demonstrated the recovery of a pulsatile BFi times series beyond ∼400 to 500 ps.^33^^,^^40^^,^^41^

In the last several years, the team at CoMind has been developing a new optical neuromonitoring platform based on parallelized time-resolved interferometric measurements at 1064 nm. We chose this wavelength because the use of 1064 nm light presents significant advantages due to the lower tissue reduced scattering coefficients, lower tissue absorption coefficients, and higher maximum permissible exposure (MPE).^28^^,^^33^ We have also worked to optimize laser performance and control, optical and optomechanical configuration, detector performance, real-time digital signal processing and analysis, and device validation methods. In this paper, we present and validate our first-generation research device—CoMind Research One (R1). This device was designed and built specifically to advance ToF-resolved measurements of BFi at pulsatile rates and to access the late photon times-of-flight necessary to ensure high brain sensitivity (and selectivity) in the adult population. The development of CoMind R1 has been guided by an extensive and novel series of Monte Carlo simulations that are described in a companion paper submitted concurrently with this work. By enabling robust, brain-selective measures of BFi, it is our intention that CoMind R1 will accelerate the clinical translation of noninvasive optical neuromonitoring technologies for the measurement of CBF.

## 2 Methods

### 2.1 CoMind Research One (R1)

**Fig. 1:**
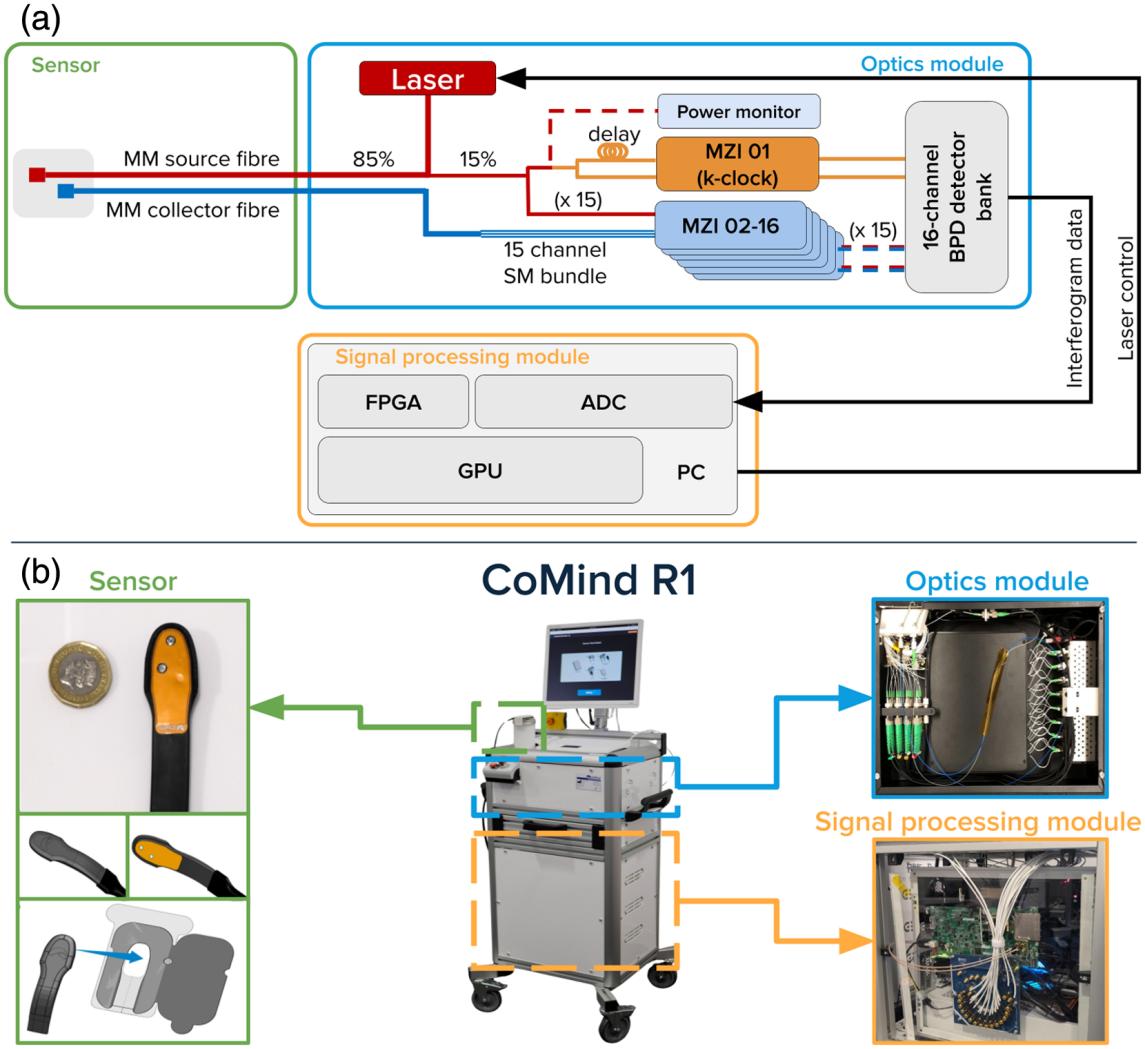
Overview of the CoMind R1 system architecture with three main modules: (i) the sensor module (green), which integrates two prism-coupled multimode optical fibers where the detection fiber is further coupled to a 15-channel SM fiber bundle in the optics module; (ii) the optics module (blue), housing a frequency-swept 1064 nm laser that is split across sample and reference arms, 16 fiber-based Mach–Zehnder interferometers (MZIs) with one of them acting as a k-clock, all connected to a 16-channel balanced photodetector (BPD); and (iii) the signal processing module (orange) with a custom ADC implemented on FPGA board and a GPU unit responsible for high-speed data acquisition and real-time digital signal processing. Panel (a) shows a schematic diagram of the system architecture, whereas panel (b) presents photographs of the CoMind R1 system with its three primary modules.

The CoMind R1 system is a 16-channel interferometric optical neuromonitoring platform, illustrated in Fig. 1. The system comprises three primary modules: (i) the optics module, which generates and routes the light for interferometric detection; (ii) the sensor, which delivers light to and collects light from the tissue or sample; and (iii) the signal processing module, which provides high-speed data acquisition and real-time processing. These modules are integrated into a portable medical cart suitable for research and clinical environments. Because of the significant advantages provided by interferometric detection, the device can be built primarily from low-cost, commonly available components. The laser is the single most expensive optical component, costing approximately $2k.

#### 2.1.1 Optics module

At the center of the CoMind R1 system is a frequency-swept distributed feedback (DFB) laser (QD Lasers, Inc., Kawasaki, Japan) with a central wavelength of 1064 nm. The laser is driven with a unidirectional sawtooth modulation waveform at a sweep rate of 200 kHz and with a sweep bandwidth of 13.2 GHz. As shown in Fig. 1, the output of the laser is split into two primary optical paths with an 85:15 splitting ratio. The 85% is routed to the sample arm and transmitted to the sensor via a 200  μm core multimode optical fiber, delivering an average of ∼60  mW to the sample. The 15% forms the reference arm, which is split again into 16 identical single-mode reference channels using a cascading series of fiber splitters. Of these 16 reference channels, one is split again to provide two critical functions: continuous laser power monitoring, and generation of a fixed-delay interference pattern that is used to monitor instantaneous optical phase—a so-called “k-clock.” The output of the k-clock is routed to a custom-built 16-channel set of balanced photodiode detectors (BPDs) bank (Thorlabs, Inc., Newton, New Jersey, United States) that consists of 16 independent pairs of InGaAs photodiodes and associated amplification electronics. The k-clock provides a feedback signal for an iterative learning control loop that is used to iteratively refine the laser-driving waveform, maximizing the linearity and phase stability of every sweep.^42^

A sawtooth sweep is particularly compatible with this control scheme because its monotonic, single-direction ramp can be corrected in real time without the asymmetries inherent to, for example, bidirectional sinusoidal sweeps. By pairing a sawtooth waveform with iterative learning control, the entire laser sweep can be efficiently exploited, yielding higher SNR and more robust performance compared with other sweeping approaches.

The backscattered light from the sample is collected via a 600  μm core multimode optical fiber, which is coupled directly to a 15-channel single-mode fiber bundle inside the optics module. The use of a multimode collector fiber, coupled to a single-mode fiber bundle, was found to improve optical throughput when compared with a single-mode to single-mode configuration. Although the exact physical basis for the observed improvement provided by our multimode-to-single-mode design is still under investigation, it may relate to the larger acceptance cone of the multimode fiber (NA≈0.39), which enables the capture of a substantially greater number of diffusely scattered photons from the tissue surface than direct single-mode collection (NA≈0.13). This may render the configuration more robust on average to imperfect coupling at the surface of the sample or skin. We cannot exclude the possibility that coupling of light from the multimode fiber into the cladding of the single-mode fibers contributes to the observed increase in optical throughput. A related result, albeit with a different MM-SM coupling mechanism, was demonstrated by Liu et al.^43^ where their “compound” fiber demonstrated improved signal quality compared with the SM detection case in both simulations and phantom experiments.

Each of the single-mode fibers within the sample arm bundle is paired with a corresponding reference fiber and coupled into a fiber-based Mach–Zehnder interferometer (MZI). A total of 15 such MZIs are implemented in parallel. Each MZI output is routed to the BPD bank, which captures the resulting temporal interference fringes for each sweep (the interferograms) while suppressing all DC and common mode noise. The interferograms captured by the BPD array encode both the photon ToFs and the dynamic speckle fluctuations caused by moving scatterers within the sample.

#### 2.1.2 Sensor

Light is delivered to and collected from the sample via an optical sensor. The sensor consists of two components: a reusable optical probe and a disposable head-mount. The optical probe integrates the 200  μm core multimode source fiber and the 600  μm core collection fiber, both of which are coupled into 2 mm glass prisms with an SDS of 10 mm. The prism coupling enables stable and consistent contact with the skin and a low-profile form factor that simplifies placement and minimizes coupling issues. The use of multimode collection simplifies sensor design and lowers costs. The reusable optical probe also incorporates a proximity sensor that supports a laser interlock mechanism that ensures the device is a class 1C laser device.

Prior to each measurement, the reusable optical probe is placed into a disposable head-mount. The head-mount includes a removable, partially reflective foil layer that is designed to permit the acquisition of a calibration measurement. This measurement is equivalent to an IRF but for the small, consistent temporal offset introduced by the optical path from the source prism surface to the foil layer and back to the detector prism surface. For simplicity, we refer to the result of this measurement as an “IRF” from this point forward. The removable foil layer makes it easy to routinely obtain an IRF for each channel prior to every measurement, which enables session- and channel-specific ToF calibration. After IRF acquisition, the reflective foil layer is simply peeled off to expose a transparent Mylar layer that lies between the reusable optical probe and the skin. Removing the foil layer also exposes a layer of medical-grade adhesive that is suitable for up to 72 h of application to human skin. The head-mount is then affixed directly to the subject’s forehead, making it possible to obtain stable and consistent contact over multiple hours of recording.

#### 2.1.3 Digital signal processing

**Fig. 2:**
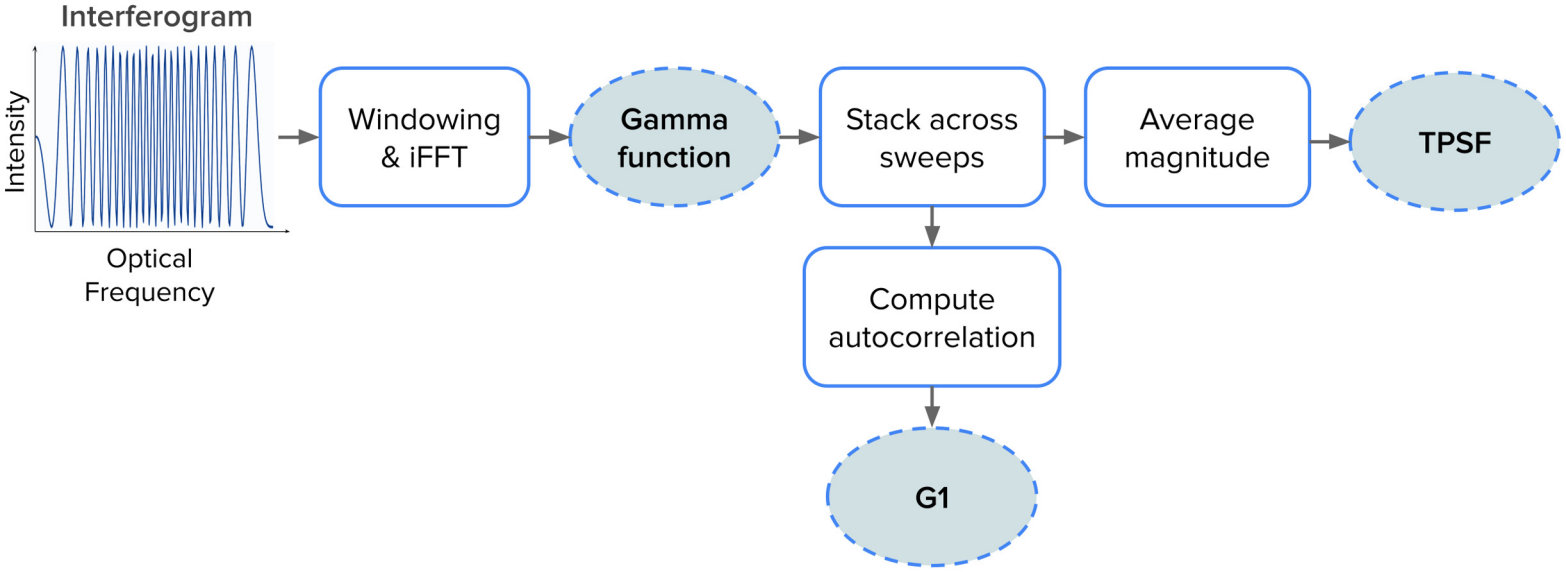
Simplified scheme of the DSP pipeline. The detected interferometric signal undergoes a series of processing steps to extract time-of-flight-resolved information. First, the raw signal is processed via iFFT to retrieve the complex Gamma function. The magnitude of it is then aligned and averaged across sweeps to yield the TPSF series, allowing downstream processing to the selected ToF gate. In the lower panel, the complex Gamma is further processed through field autocorrelation ($G_{1}(\tau )$) computation, denoising, and normalization steps to obtain the normalized electric field autocorrelation function, $g_{1}(\tau )$, which decay rate reflects dynamic properties of the tissue.

The analog interferometric signals from the BPD bank are digitized by a custom 16-channel, 16-bit analog-to-digital converter (ADC) operating at 100 MHz and implemented on a field-programmable gate array (FPGA) that processes over 25 Gb of raw interferogram data per second. The interferograms are then passed to the GPU-enabled digital signal processing (DSP) pipeline that runs in real time, simultaneously with ongoing data acquisition. This pipeline is outlined in Fig. 2. First, each interferogram is subject to an inverse Fourier transform (iFFT) to recover the complex temporal field, commonly called the mutual coherence (Gamma) function.^29^ For every 40,960 consecutive sweeps (equivalent to ≈200  ms of integration time or ≈5  Hz of acquisition rate), the magnitude of the Gamma functions is averaged to obtain a single TPSF.

For each ToF gate and channel, a block of 8192 with a stride of 4096 complex Gamma functions is stacked and autocorrelated to obtain the electric-field temporal autocorrelation function, $G_{1}(\tau )$, where τ represents the autocorrelation lag time; these $G_{1}(\tau )$ traces are stored individually at an effective acquisition rate ≈50  Hz. Because of the system’s finite IRF, each ToF gate represents a temporal window, not a single discrete photon arrival time. Accordingly, signals attributed to a particular ToF delay (e.g., 0.2 ns) effectively reflect photon arrival times from the ToF gate as wide as the full width at half maximum (FWHM) of the IRF centered at a given ToF delay. Throughout this work, ToF gates are treated as overlapping time windows centered at a given ToF delay, in line with the effective IRF-limited resolution of the system. For all ToF delay calculations, the zero ToF point is set at the peak of the IRF.

#### 2.1.4 Data post-processing

To reduce noise and enhance signal quality, custom denoising algorithms are applied to $G_{1}(\tau ,\mathrm{ToF})$ prior to normalizing, channel-combination, and fitting.

Our denoising approach is based on multiset canonical correlation analysis (mCCA) as described by Parra et al.^44^ and de Cheveigné et al.,^45^ adapted here to multichannel $G_{1}$ data. Briefly, the mCCA method extends canonical correlation analysis (CCA) to more than two datasets. Given n multidimensional datasets derived from the same system, the algorithm identifies projections that maximize correlations between these sets.

In our application, the $G_{1}s$ for a given ToF provided by each of our 15 detection channels provide n=15 multidimensional datasets, with those dimensions being autocorrelation lag and sample time. Each ToF is treated independently, thereby preventing information leakage. A calibration step is performed using 500 samples (10 s) of $G_{1}$ data, from which cross-channel covariance matrices are estimated and linear projection matrices are determined. The resulting components are ranked based on how consistently each component is expressed across the datasets as quantified by the eigenvalues of the mCCA decomposition. The first four components are retained and back-projected to reconstruct denoised $G_{1}s$ for a given ToF. This approach therefore rejects noise that is inconsistent across datasets (i.e., channels) and retains common features, which is expected to include features driven by the optical characteristics of the sampled object. A Bland–Altman plot for the dataset with 25 subjects comparing denoised versus nondenoised data is provided in Fig. S1 in the Supplementary Material, demonstrating that this approach introduces a negligible bias in the recovered autocorrelation decay rate.

For consistency across time-of-flight gates, the normalized field autocorrelation is then obtained as:

$$g_{1}(\tau ,\mathrm{ToF})=\frac{G_{1}(\tau ,\mathrm{ToF})}{G_{1}(\tau_{1},\mathrm{ToF})},$$ (1)

where $\tau_{1}=5\;\mu s$. The zero-lag value $G_{1}(\tau =0)$ is excluded from the analysis.^40^ This simple normalization removes constant scaling factors related to interferometric visibility while preserving the true temporal decorrelation behavior associated with photon dynamics and flow.

According to diffusing wave spectroscopy (DWS), the decay rate of the electric field autocorrelation function at a given ToF, ξ(ToF), can be expressed as:

$$\xi (\mathrm{ToF})=2k^{2}\alpha D_{b}\mu_{s}^{\prime }v\mathrm{ToF},$$ (2)

where k is the optical wavenumber, $\alpha D_{b}$ is the effective diffusion coefficient of moving scatterers (proportional to blood-flow-related motion), $\mu_{s}^{\prime }$ is the reduced scattering coefficient, and v is the speed of light in the medium.

The normalized autocorrelation functions $g_{1}(\tau )$ are then truncated such that either all points with $g_{1}(\tau )>0.5$ and τ<100  μs, or the points associated with the first five lags, (whichever is greater), are fitted with a single-exponential decay model:

$$g_{1}(\tau )=e^{-\xi \tau },$$ (3)

where ξ is the $g_{1}$ decay rate parameter in Hz and, according to Eq. (2), is directly proportional to $\alpha D_{b}\mu_{s}^{\prime }$.^29^^,^^41^ Therefore, any change in either the dynamic component ($\alpha D_{b}$) or the static scattering properties ($\mu_{s}^{\prime }$) will alter the measured decay rate. In biological tissue, both parameters can vary across depth (e.g., between scalp and cortex) and over time (e.g., due to physiological modulation or activation). As a result, interferometric and diffuse correlation measurements inherently access the combined parameter $\alpha D_{b}\mu_{s}^{\prime }$ rather than absolute cerebral blood flow in isolation. This coupling represents a limitation shared by all diffuse optical methods, not solely specific to CoMind R1. It highlights the importance of controlling or estimating optical properties when interpreting flow-related parameters and motivates hybrid approaches that combine time-resolved interferometry with techniques capable of retrieving optical properties, or with independent modalities for multimodal calibration.

For each time-of-flight gate, a time series of ξ values is extracted, and the relative BFi (rBFi) at each ToF, at a given time point (t) is defined as:

$$r\mathrm{BF}i(t)=\frac{\xi (t)}{\left\langle \xi (t_{0})\right\rangle },$$ (4)

where $\left\langle \xi (t_{0})\right\rangle$ denotes the temporal mean of ξ over a selected baseline period. This normalization step allows for direct comparison of relative BFi changes across subjects, protocols, and ToF gates.

This entire denoising-and-fitting routine can be executed either offline or online in real time. When operating in real time, CoMind R1 generates a continuous BFi time series at a selected ToF and provides real-time feedback on data quality via a custom pulse-quality metric (see Sec. 2.3.1).

### 2.2 *In Vitro* Validation Protocols

The primary objective of this work is to demonstrate and validate the performance of CoMind R1 in terms of signal quality, temporal stability, and depth sensitivity. To achieve this, a series of validation studies that include both *in vitro* and *in vivo* experiments was conducted.

Note that CoMind R1 is inherently insensitive to ambient light due to its interferometric detection strategy, and an experimental demonstration of this has been reported previously^46^ and is not repeated here. However, it is worth highlighting that all of the experimental data presented herein were collected in a well-lit laboratory environment without applying additional optical isolation over the sensor or otherwise attempting to minimize the ingress of ambient light.

#### 2.2.1 Long-duration homogeneous phantom

**Fig. 3:**
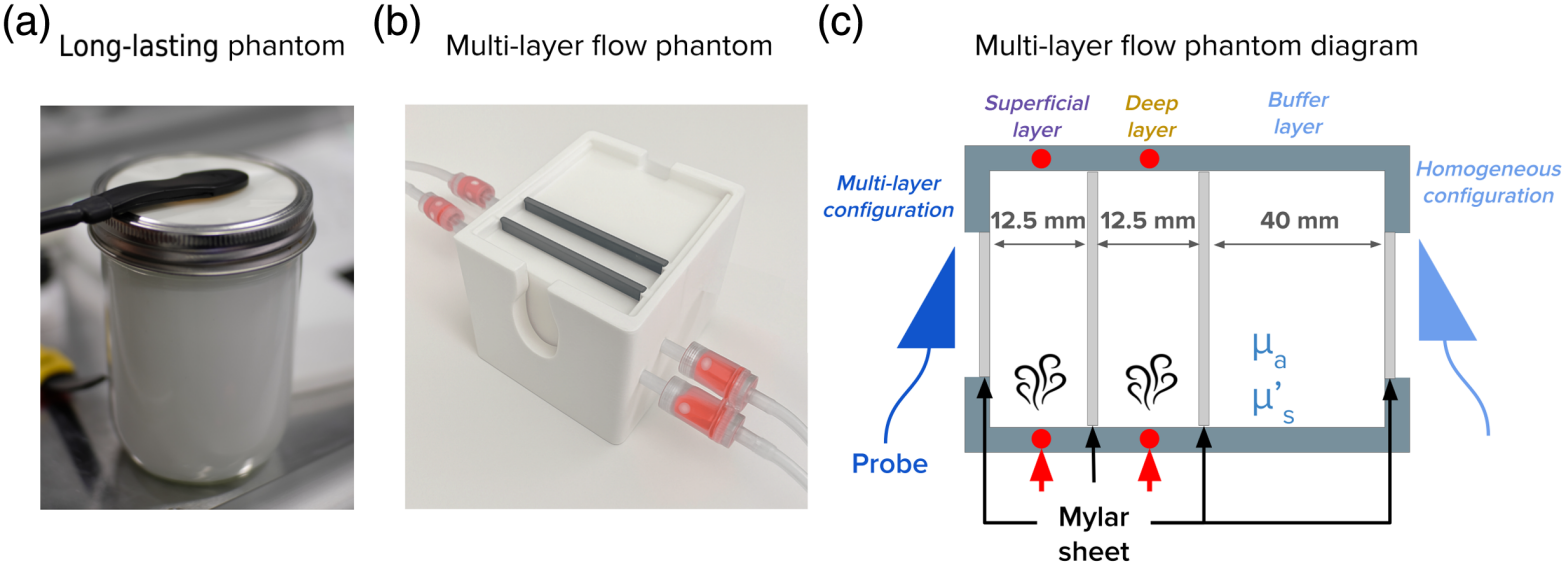
Images of the experimental phantoms used for system validation. (a) The homogeneous, long-lasting liquid phantom is used to assess device stability; (b) the multilayer dynamic flow phantom that is designed to examine depth sensitivity; and (c) a schematic diagram of the multilayer dynamic flow phantom, illustrating how recordings are made in either a homogenous configuration or multilayer configuration depending on probe location.

A long-lasting homogeneous phantom [Fig. 3(a)] was constructed and used to assess the fundamental performance and longitudinal stability of CoMind R1. This intralipid-based liquid phantom^47^ was designed to replicate the reduced scattering ($\mu_{s}^{\prime }$) and absorption coefficients ($\mu_{a}$) of tissue at 1064 nm ($\mu_{s}^{\prime }=7.4\;{\mathrm{cm}}^{-1}$ and $\mu_{a}=0.12\;{\mathrm{cm}}^{-1}$ respectively). The phantom was enclosed in a pressure-sealed glass jar with a 75  μm Mylar optical window forming the upper surface to provide an optical coupling interface for the sensor. A preservative—diazolidinyl urea—was added to the Intralipid solution to increase longevity without affecting the optical properties. Each experiment was conducted in a temperature-controlled room to provide consistent environmental conditions throughout the measurement period.

A 24-h-long recording was acquired with CoMind R1 applied to this homogeneous phantom. To assess signal quality and stability, the mean and standard deviation (STD) of the $g_{1}$ decay rate as a function of ToF was calculated. To evaluate potential signal drift over the 24-h recording period, a linear regression analysis was applied to the mean signal amplitude calculated within nonoverlapping 30-min windows at every ToF gate up to 1.2 ns. A linear regression model was then fitted to the windowed mean values as a function of time. The slope of the regression line, along with its statistical significance, was used to quantify and evaluate the presence of drift over the full 24-h duration of the recording.

#### 2.2.2 Multilayer flow phantom

The depth sensitivity of the CoMind R1 system was evaluated using a multilayer dynamic optical phantom specifically designed to replicate dynamic flow variations within multiple highly scattering tissue layers. The phantom comprises three distinct compartments, each separated from the neighboring layer(s) by a 75  μm Mylar membrane, allowing optical transmission between layers while maintaining the structural integrity of the phantom [Figs. 3(b) and 3(c)]. The thicknesses of the superficial (first) and deep (second) layers (which were designed to mimic the superficial and brain tissues, respectively) were fixed at 12.5 mm, corresponding to the median scalp-to-cortex distance reported for adults over the pre-frontal lobe.^48^ The third layer has a thickness of 40 mm and serves as a buffer layer, ensuring appropriate boundary conditions are maintained. The phantom is constructed with two Mylar windows: one window providing direct optical access to the buffer layer, which allows the same phantom to be used to provide measurements of an effectively homogeneous medium (the “homogeneous configuration”); and the other one providing optical access to the superficial layer and, through it, to the deep layer (the “multilayer” measurement configuration). The superficial and deep layers are connected to independent peristaltic pumps that can drive the intralipid phantom solution through each of those layers at a selectable rate.

All compartments were then filled with an Intralipid-based fluid with controlled optical properties matching biological tissue at a wavelength of 1064 nm ($\mu_{s}^{\prime }=7.4\;{\mathrm{cm}}^{-1}$, $\mu_{a}=0.12\;{\mathrm{cm}}^{-1}$). The optical sensor was positioned on the optical window of the superficial layer, i.e., in the multilayer configuration. The independent peristaltic pumps were then used to induce variable flow rates in the superficial (i.e., “scalp” and “skull”) and the deep (i.e., the “brain”) layers. This configuration allowed systematic characterization of the system’s sensitivity to deep-layer flow dynamics under conditions where the flow in both layers was representative of the tissues of the human head.

To determine physiologically relevant flow rates for the phantom, we leveraged the dataset acquired from N=25 healthy adult volunteers at rest using the CoMind R1 device. Specifically, the median and range of *in vivo* systolic and diastolic $g_{1}$ decay rates were calculated as a function of photon ToF. Further details about this dataset and its acquisition can be found in Sec. 2.3.

For the superficial phantom layer, pump flow rates were empirically identified to approximate the diastolic and systolic $g_{1}$ decay rates observed *in vivo* at ToF≤0.2  ns; the assumption being that at this early ToF, the *in vivo* measurements will be dominated by the superficial tissues. Having established these low and high superficial flow rate conditions, the pump for the superficial layer was set to the high (systolic) flow case, and the deep layer pump flow rate was systematically increased until the decay rate at late ToF (∼1  ns) was approximately equal to the systolic median decay rate observed *in vivo* at that late ToF. This allowed us to establish an upper bound for the flow rate of the deep-layer pump. The lower bound was then set to match the low (diastolic) flow rate determined for the superficial layer. Through this process, it was possible to identify physiologically reasonable lower and upper bounds on flow in both the superficial and deep layers of the phantom.

Once these boundary flow rates had been determined, the identified range of deep-layer flow rates was stepped through while the superficial layer flow rate was set at either the low (diastolic) or high (systolic) condition. This phantom, therefore, allowed us to evaluate the capacity of CoMind R1 to resolve deep-layer flow dynamics at physiological scales, under conditions of high or low superficial flow, while maintaining appropriate absorption and scattering coefficients and anatomically relevant layer thicknesses.

Although the diffusion model assumes a homogeneous medium, it can still approximate motion dynamics in a layered system. In the present phantom, each layer exhibits distinct flow velocities, and the detected field autocorrelation represents a weighted mixture of their motion statistics, well approximated by a single-exponential $g_{1}$ decay. The fitted decay rate then serves as an effective motion parameter encompassing both diffusive and flow-driven dynamics.

To estimate the ToF at which CoMind R1 first became sensitive to the “brain” layer in this multilayer phantom, a sliding-window linear regression analysis was applied to the multilayer dynamic phantom data. Using a five-point window advancing in ToF one bin at a time, the local gradient of $g_{1}$ decay rate versus ToF was computed by linear least-squares fitting. The five-point window width was chosen empirically as the smallest interval that yielded stable slope estimates while preserving local structure in the ToF-dependent trends. For each window position, the regression slopes for each deep-layer flow condition were compared with those from the homogeneous (minimal-flow) reference case using two-sample z-tests (threshold: |z|>10). The last point of the earliest ToF window at which these gradients differ is interpreted as the point where the measurement first becomes meaningfully sensitive to the deeper layer, i.e., a cut-off point where the deeper layer measurably alters the gradient of the detected signal, even though its contribution to the overall decay rate may still be small.

#### 2.2.3 Homogeneous phantom with varying scattering

The aim of this experiment was to evaluate how signal quality (quantified by the standard deviation of the $g_{1}$ decay rate as a function of ToF) varies with increasing decay rate. To achieve controlled changes in the $g_{1}$ decay rate, the reduced scattering coefficient was systematically adjusted.

The optical sensor was positioned on the optical window of the buffer layer of the multilayer optical phantom [Fig. 3(c)], i.e., in the homogeneous configuration. The absorption coefficient of the buffer layer was fixed at $\mu_{a}=0.12\;{\mathrm{cm}}^{-1}$, whereas the reduced scattering coefficient was varied systematically across six conditions: $\mu_{s}^{\prime }$ = (5, 10, 20, 30, 40, and 50) ${\mathrm{cm}}^{-1}$. Each phantom condition was achieved by adjusting the concentration of Intralipid to achieve the target reduced scattering coefficient value.

For each scattering condition, a 2-minute-long recording was acquired, and $g_{1}$ decay rate values were computed to assess device measurement noise as a function of decay rate and ToF. The experiment was conducted at room temperature (∼22°C), and sufficient settling time was provided to ensure the phantom was in thermal equilibrium with the environment before acquisition.

### 2.3 *In Vivo* Validation Protocols

To complement the *in vitro* validations and assess system performance under real physiological conditions, we conducted a series of *in vivo* experiments on healthy adult volunteers. These experiments were designed to evaluate signal quality and depth sensitivity in real anatomical and physiological cases. All *in vivo* studies were conducted at CoMind’s laboratories in London, UK. Each experimental protocol was approved by an in-house ethical review process that incorporated an independent external expert review. Prior written informed consent was obtained from each volunteer.

#### 2.3.1 *In vivo* pulsatile signal quality distribution

As the capacity to resolve robust pulsatile BFi is a key goal of CoMind R1, we recruited 25 healthy adults (age 34±8 years; 16 males, 9 females) to perform a simple at-rest measurement to assess pulse quality as a function of ToF using the CoMind R1 system. Participants were seated in a comfortable sitting position with minimal movement and external stimulation.

The CoMind R1 sensor was placed on the forehead at approximately FP1 according to the 10-20 EEG system. Prior to sensor placement, the participant’s forehead was cleaned in the intended sensor area using a sterile alcohol wipe. The midpoint between the source and detector was aligned with the center of the subject’s eye socket while the lower edge of the sensor was positioned ∼2  cm above the eyebrow line. A 2-minute-long recording was acquired, and rBFi values were computed as a function of ToF for each subject.

To quantify signal quality at each ToF, we developed the pulse variance ratio (PVR)—a metric designed to assess the consistency of pulsatile waveforms in time-series data. For each ToF gate and subject, a train of 10 consecutive cardiac pulses was segmented from the rBFi time-series, z-scored, and resampled to a common time axis. The expectation value over time of the variance across pulses ($\left\langle \sigma_{\text{across}}^{2}\right\rangle$, i.e., the between-pulse variability) and the average variance across time within each pulse ($\left\langle \sigma_{\text{within}}^{2}\right\rangle$, i.e., the intra-pulse variability) were then computed:

$$\langle \sigma_{\text{within}}^{2}\rangle =\frac{1}{N_{\text{pulses}}}\overset{N_{\text{pulses}}}{\underset{i=1}{\sum }}\sigma_{\text{within},i}^{2},$$ (5)

$$\langle \sigma_{\text{across}}^{2}\rangle =\frac{1}{N_{\text{samples}}}\overset{N_{\text{samples}}}{\underset{j=1}{\sum }}\sigma_{\text{across},j}^{2}.$$ (6)

The PVR was then defined as:

$$\mathrm{PVR}=1-\frac{\left\langle \sigma_{\text{within}}^{2}\right\rangle }{\left\langle \sigma_{\text{across}}^{2}\right\rangle }.$$ (7)

A PVR value of 1 corresponds to a train of perfectly identical pulses, lower values indicate greater variability in pulse morphology, and zero would indicate the signal is equivalent to noise. Note that due to physiological variation in pulse shapes beat-to-beat, even if the measurement noise was zero, a PVR of exactly 1 would not be expected. This PVR metric allows for an objective comparison of pulsatile BFi signal quality across ToF gates and subjects without making any assumptions about pulse morphology or pulse rate.

Note that the PVR metric was selected not as a direct measure of system SNR, but as a robust indicator of the temporal stability and reproducibility of the pulsatile component in the BFi signal across consecutive cardiac cycles. Because PVR reflects pulse stability, it is affected by subject-specific physiological characteristics such as pulsatility index. As a result, recordings of two different subjects with identical SNR could yield different PVR values. However, given that our goal is to demonstrate pulsatile measures of CBF, the presence of a robust pulsatile signal at a given ToF represents a necessary (but not sufficient) condition for the success of any given measurement.

An empirically defined threshold PVR value of 0.6 was used to define the maximum ToF at which a subject’s signal was considered high quality, referred to as the “Corner ToF.”

#### 2.3.2 Visual stimulation

To demonstrate the depth sensitivity of CoMind R1 *in vivo*, we employed a visual stimulation paradigm that is known to provide a reliable cortical functional hyperemic response with minimal evoked systemic haemodynamics.^49^

We recruited 16 healthy subjects, aged 35±9 years (9 males, 7 females), for visual stimulation experiments. The visual stimulation paradigm consisted of 15 trials of 13 s of left hemi-field reversing checkerboard stimulation at 10 Hz mixed with 15 control trials of 13 s of a luminance-matched blank screen, in a random order. Each trial was followed by 20 s of rest. To examine changes in CBF due to the visual stimulus, rBFi values were normalised using the mean of the 10 s of data prior to the start of each trial as a baseline. Cardiac pulsatile components were attenuated by subjecting the rBFi signal to a low-pass filter with a 0.5 Hz cutoff frequency. The probe was centered over the right primary visual cortex, guided by the EEG 10-20 system. For this protocol, straight source and collection optical fibers were used instead of the prism-coupled optics described above to facilitate placement over the occipital region through hair.

Together with CoMind R1, subjects were simultaneously monitored with a noninvasive continuous arterial blood pressure (ABP) monitor (Finapres Nova, Finapres Medical Systems BV, Netherlands). Heart rate was extracted from ABP signal using NeuroKit2,^50^ which detects systolic peaks, computes inter-beat intervals, converts them to instantaneous heart rate, and interpolates the resulting values to produce a continuous heart rate time series.

To assess the significance of the responses to visual stimulation, we performed statistical testing on the difference between stimulus and control trials. For each participant, the mean rBFI change during stimulus minus control trials was computed over the 5 to 20 s post-stimulus onset window, and the resulting distribution of differences across the 16 subjects was tested against zero using a one-sample t-test (two-tailed). The same approach was applied to ABP and heart rate signals. Statistical significance was defined as p<0.05. The results are reported as mean ± standard error of the mean (SEM).

## 3 Results

### 3.1 *In Vitro* Validation

#### 3.1.1 Long-duration homogeneous phantom

**Fig. 4:**
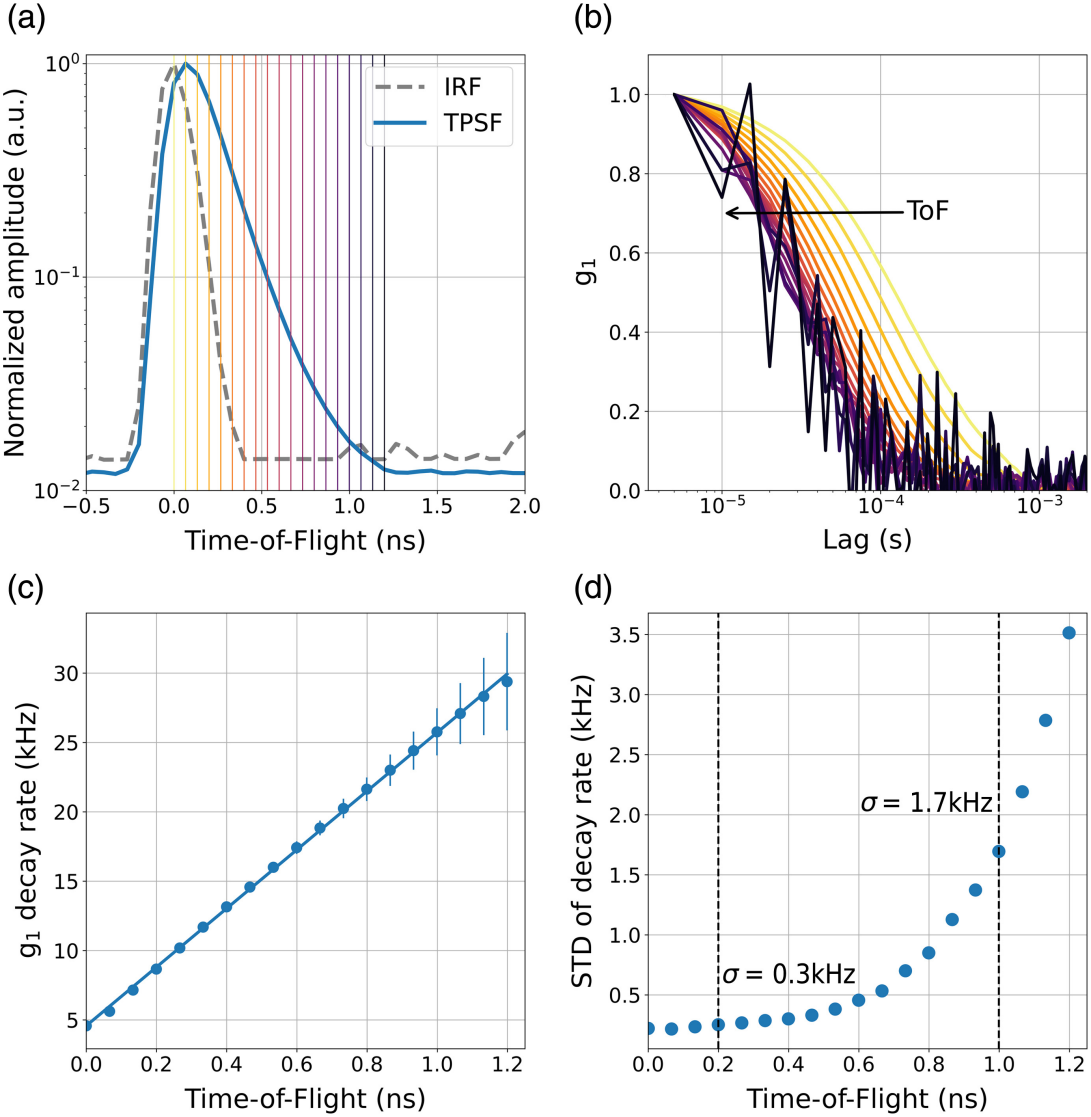
Longitudinal 24-h homogeneous phantom measurement results: (a) an example IRF (dashed grey line) and TPSF (solid blue line), with ToF gates marked with vertical lines; (b) channel averaged $g_{1}$ curves for the ToF gates defined in (a); (c) $g_{1}$ decay rate versus ToF shown as mean and standard deviation (error bars) recorded over the full 24 h; (d) standard deviation of $g_{1}$ decay rate as a function of ToF with dashed vertical lines marking σ=0.3  kHz at 0.2 ns and σ=1.7  kHz at 1 ns.

Figure 4 presents the results of a 24-h longitudinal measurement on the long-lasting tissue-mimicking phantom, designed to assess CoMind R1’s temporal stability and general performance. Figure 4(a) shows the IRF (dashed), acquired in 2 s using the reflective layer within the custom head-mount described above. The IRF has a FWHM of 120±14  ps. Figure 4(a) also shows a representative TPSF acquired in 0.2 s (acquisition rate 5 Hz), with selected ToF gates indicated by vertical lines. Figure 4(b) displays the 15-channel-averaged $g_{1}(\tau )$ curves for each ToF gate, acquired at 50 Hz with no temporal averaging, showing a consistent and expected trend of faster decay at later ToF gates. Figure 4(c) shows the mean extracted $g_{1}$ decay rates versus ToF, showing a monotonic and approximately linear increase in decay rate with increasing ToF. The standard deviation (σ) of the $g_{1}$ decay rate measured at a sampling rate of 50 Hz is 0.3 kHz at a ToF of 0.2 ns and 1.7 kHz at a ToF of 1 ns. The regression analysis showed no observable systematic drift in the signal over the 24-h recording period with p>0.05 at every ToF gate.

#### 3.1.2 Homogeneous phantom with varying scattering

**Fig. 5:**
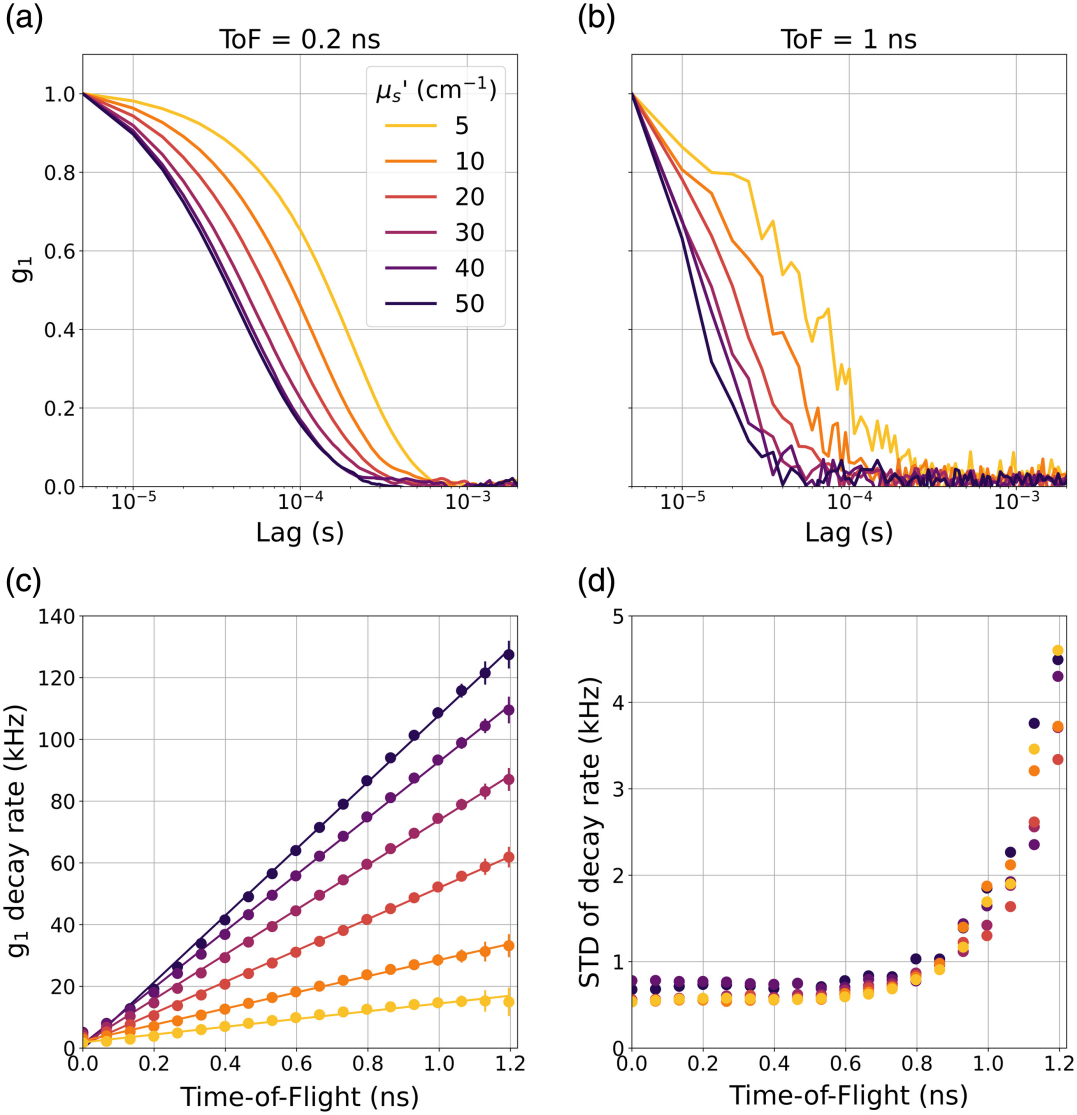
Homogeneous phantom with varying scattering and fixed absorption ($0.12\;{\mathrm{cm}}^{-1}$). (a) $g_{1}$ curves for different reduced scattering coefficient values at ToF = 0.2 ns and (b) ToF = 1 ns; (c) the $g_{1}$ decay rate as a function of ToF for different reduced scattering coefficient values, demonstrating how the slope of $g_{1}$ decay rate versus ToF changes with increasing scattering. (d) The standard deviation of the $g_{1}$ decay rate (measured at 50 Hz) as a function of ToF for all values of reduced scattering coefficient.

Figure 5 illustrates the effect of varying the reduced scattering coefficient on the $g_{1}$ decay rate and its standard deviation as a function of ToF. The $g_{1}$ decay rate increases smoothly and monotonically with both ToF and reduced scattering coefficient values. This behavior reflects the expected relationships between scattering-induced decorrelation and photon path length. The experimental range of reduced scattering coefficients provided a $g_{1}$ decay rate ranging from ~15 to 100 kHz at 1 ns [Fig. 5(a)]. Note that the standard deviation of CoMind R1 measurements (at a sampling rate of 50 Hz) not only remains in the range of 1 to 2 kHz at ToFs beyond 1 ns but is also largely independent of $\mu_{s}$’ and therefore of decay rate up to at least ∼100  kHz.

#### 3.1.3 Multilayer flow phantom experiment

**Fig. 6:**
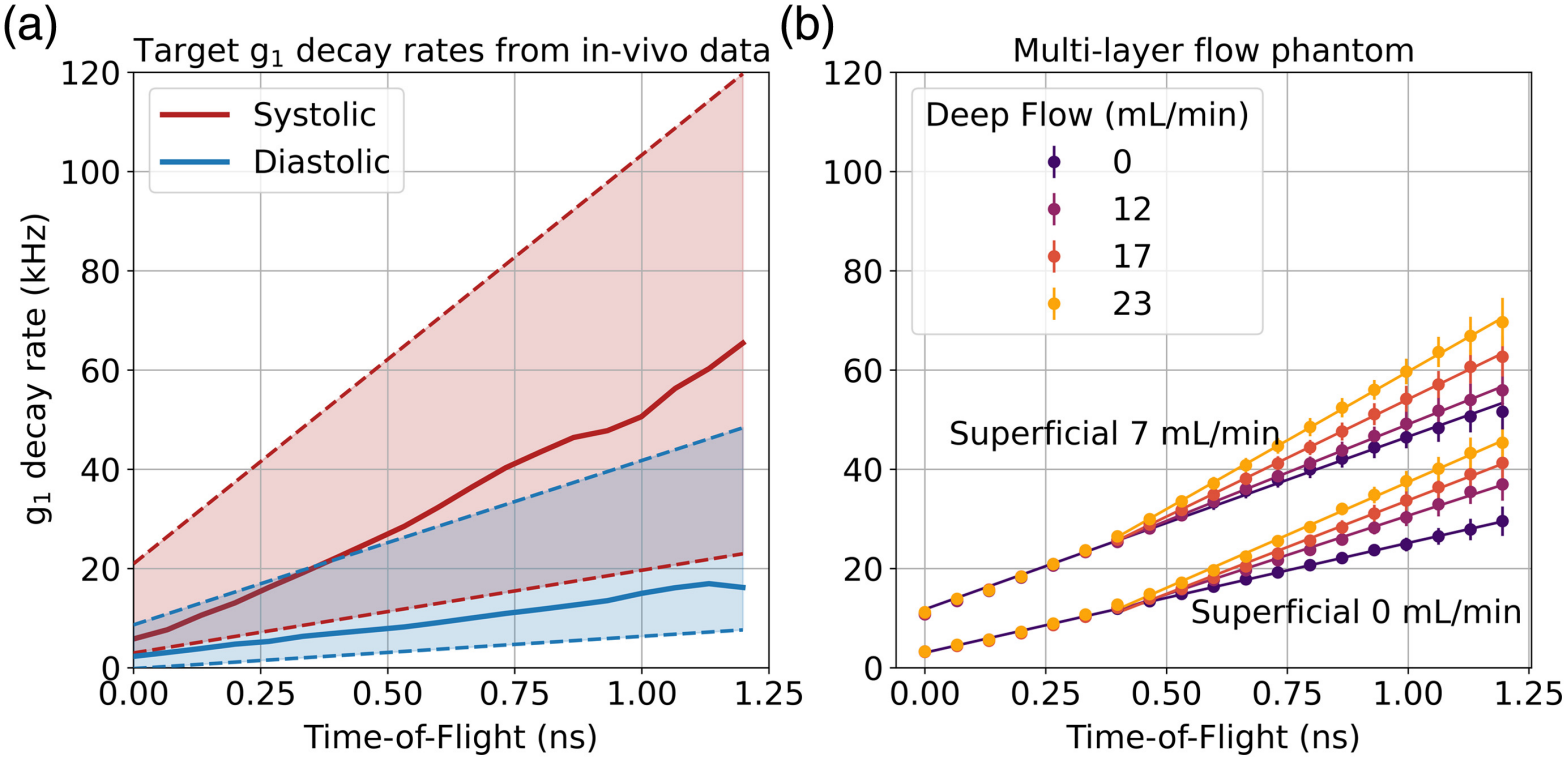
(a) Target systolic and diastolic ranges (shaded areas) of $g_{1}$ decay rate versus ToF derived from *in vivo* forehead measurements from 25 subjects. Solid lines represent medians, and the shaded areas represent the spread of the measured decay rates for both cases. Note that the mean decay rate at 1 ns across the cardiac cycle and across all subjects was 28 kHz. (b) The $g_{1}$ decay rates as a function of ToF for the multilayer flow phantom for two different superficial layer flow rates (0 and 7  mL/min) and four different deep layer flow rates from 0 to 23  mL/min.

Figure 6(a) depicts the median and the linearized range of decay rate values observed at diastole and systole in the N=25 healthy subjects. Using this data as a guide, low (diastolic) and high (systolic) pump flow rates were identified for the superficial layer as 0  mL/min (i.e., the intralipid solution without pump-induced flow) and 7  mL/min, respectively. The upper bound on the deep layer pump flow rate was found to be 23  mL/min (3.3 times that of the superficial layer).

Figure 6(b) illustrates the measured $g_{1}$ decay rates as a function of ToF for both the low and high superficial flow rates and the full range of deep flow rates. It is first evident that two distinct sets of decay rates are formed depending on the flow rate in the superficial layer. Introducing a systolic-equivalent superficial layer flow rate (7  mL/min) introduces a large offset in $g_{1}$ decay rate across all ToFs and affects the gradient at early ToFs. Examples of the autocorrelation curves and their respective fits for the four most extreme cases are shown in Fig. S2 in the Supplementary Material.

In both low and high superficial flow cases, the measured $g_{1}$ decay rates for the different deep layer flow cases are indistinguishable at early ToF (until at least ∼0.3  ns), providing evidence that the signals acquired at these ToFs are entirely driven by the characteristics of the superficial layer.

Increasing the flow rate in the deep layer from 0 to 23  mL/min results in a systematic increase in $g_{1}$ decay rates at later ToFs for both the low and high superficial flow cases. At, e.g., 1 ns, the varying deep layer flow rates induce a range of decay rates spanning ∼15  kHz. The decay rate values associated with each deep layer pump setting are distinct from one another at late ToF, even in the presence of high superficial flow. Note that error bars depict the measurement standard deviation (at a sampling rate of 50 Hz), with the maximum observed standard deviation at 1 ns equal to 2.6 kHz. Note that this is markedly higher than the equivalent value for the homogeneous phantom [Fig. 5(d)], suggesting that the dynamic phantom itself introduces noise due to pump flow rate variation or other sources.

Furthermore, changes in the slope of $g_{1}$ decay rate versus ToF are apparent, even at relatively early ToF. The sliding-window linear regression analysis determined that the earliest ToF at which the deep layer significantly impacts the gradient of the measured $g_{1}$ decay rate versus ToF was 0.39 ns. Based on this, Fig. 6(b) includes piecewise linear fits to the $g_{1}$ decay rate values for the ToF ranges before and after this change point, which highlight the evident changes in gradient.

To quantify sensitivity to deep-layer flow, the relative change in $g_{1}$ decay rate at 1.2 ns was calculated for both superficial flow conditions. When superficial flow was 0  mL/min, increasing deep-layer flow from 0 to 23  mL/min resulted in an increase in $g_{1}$ decay rate from 25 to 40 kHz (or 60%), with a strong linear relationship between $g_{1}$ decay rate and pump flow rate (Pearson R=0.997, p<0.01). With a superficial flow of 7  mL/min, the same increase in deep-layer flow produced an increase in $g_{1}$ decay rate from 49 to 66 kHz (35%) and also showed a strong linear dependence (R=0.99, p<0.01). This attenuation of the response reflects a significant contribution from superficial dynamics affecting response amplitude.

### 3.2 *In Vivo* Validation

#### 3.2.1 *In vivo* pulsatile signal quality distribution

**Fig. 7:**
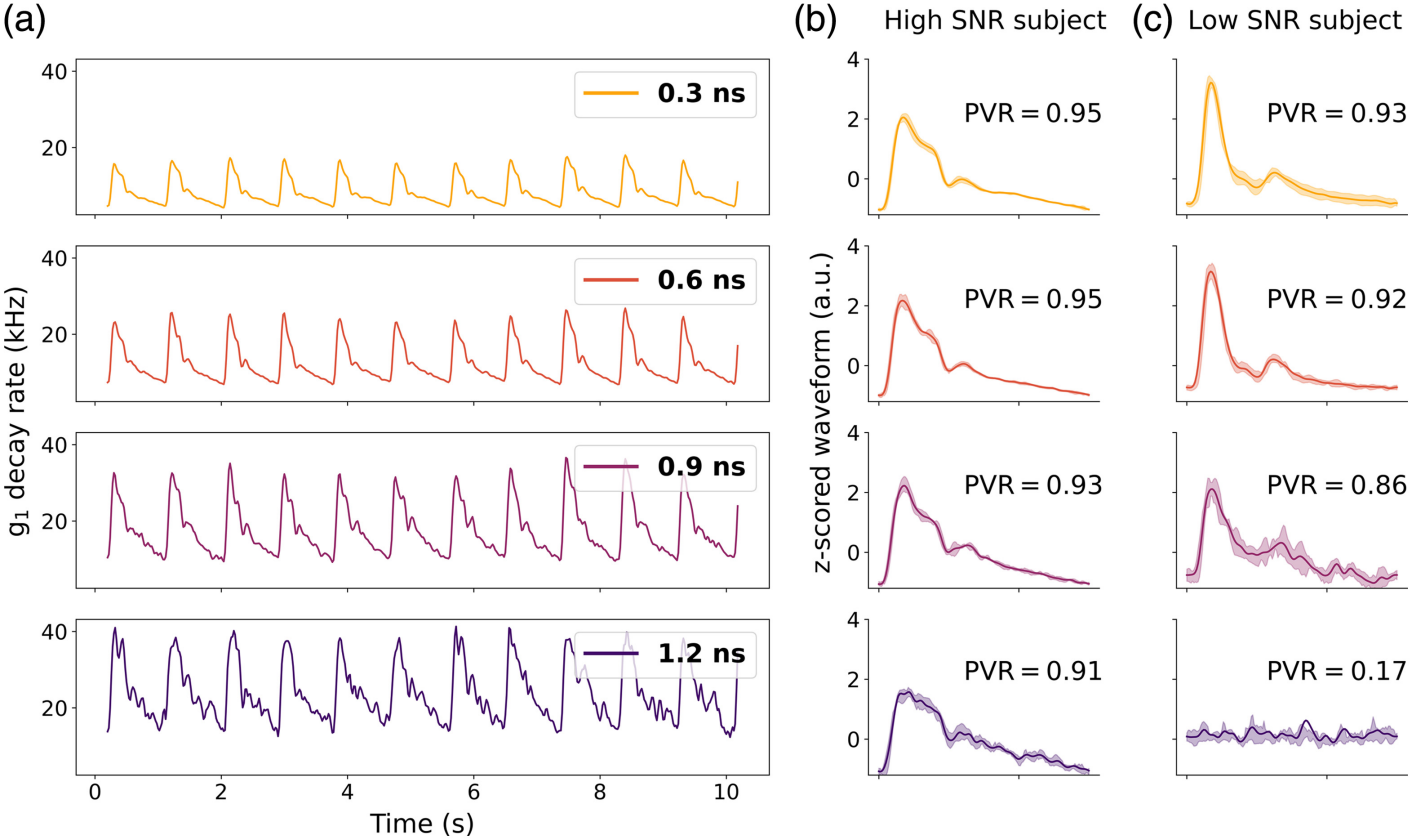
Examples of pulsatile time trace at different ToFs for one subject are shown in panel (a), and examples of mean (solid) and std (shaded) of averaged pulses corresponding to different PVR values at different ToFs for two representative subjects are shown in panels (b) and (c).

The *in vivo* signal quality assessment was conducted to evaluate the performance and reliability of the system across a diverse group of healthy adult participants. To demonstrate an example of low-noise, pulsatile blood flow measured with CoMind R1, Fig. 7(a) shows representative rBFI time traces at ToFs ranging from 0.3 to 1.2 ns in a single subject. Note that the pulsatile features are preserved across all ToFs, although increased noise is apparent at later ToFs.

**Fig. 8:**
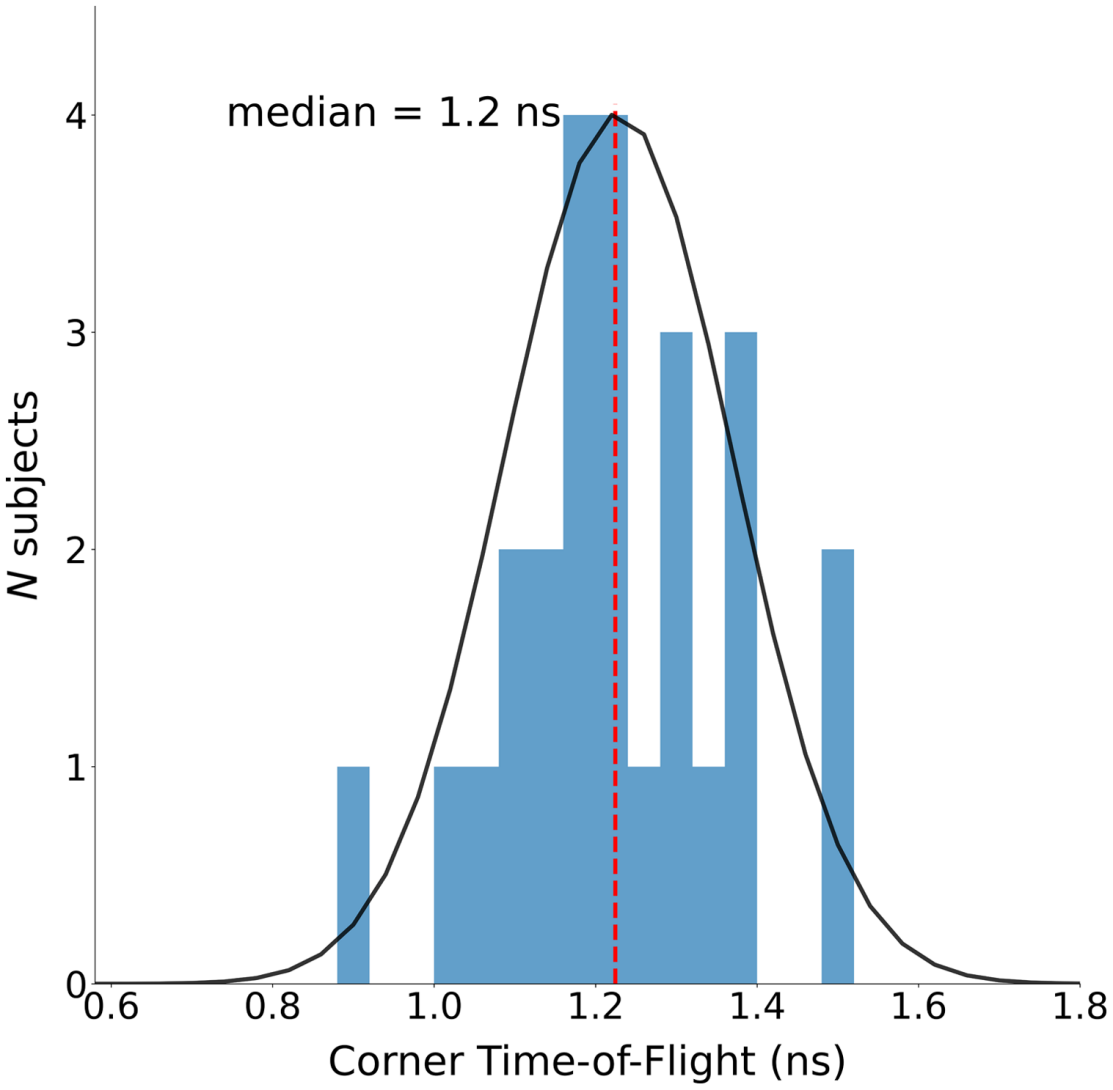
Distribution of the Corner ToF values across a cohort of 25 subjects. The corner ToF, defined as the latest time gate at which high-quality pulsatile signals are reliably detected with a median corner ToF of 1.2 ns (indicated by the red dashed line).

Figure 7(b) presents averaged z-scored waveforms from two individuals with differing signal quality, as quantified by the pulse-to-variance ratio (PVR). These data illustrate how this metric reflects the fidelity of the pulsatile waveform. The distribution of Corner ToFs across the cohort is shown in Fig. 8. The vast majority of subjects demonstrated high PVR values beyond 1 ns, with a median corner ToF of 1.2 ns. The subject with the worst signal quality yielded a corner ToF of 0.9 ns. These findings indicate that the CoMind R1 system is capable of reliably acquiring low-noise pulsatile blood flow signals at ToFs exceeding 1 ns in the vast majority of individuals.

Note that based on these results, a threshold corner ToF of 0.9 ns (i.e., a PVR>0.6 at ToF = 0.9 ns) was established as a signal-quality threshold to guide sensor attachment for the visual stimulation experimental protocol described below. This threshold was used in real time to guide the experimentalist in optimizing probe positioning and optical coupling.

#### 3.2.2 Visual stimulation

**Fig. 9:**
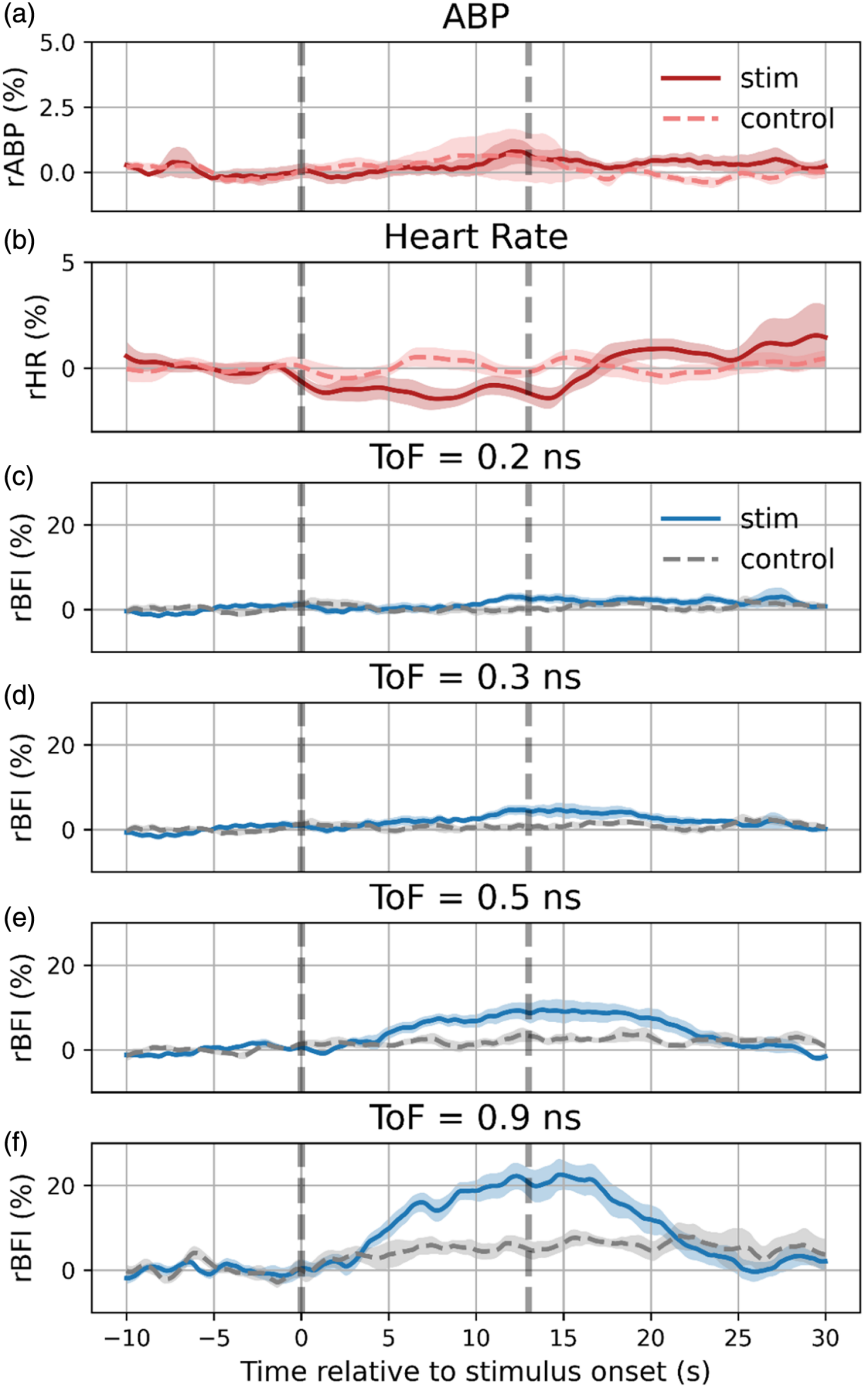
Group-averaged responses during the visual stimulation protocol for 16 subjects. (a), (b) rABP and rHR traces for stimulation (solid dark red line) and control (dashed red line) trials; (c) mean rBFi responses at the early time-of-flight (ToF = 0.2 ns), showing no significant difference between stimulation and control trials; (d) mean rBFi responses at the first time-of-flight with a significant response (ToF = 0.3 ns); (e), (f) intermediate (ToF = 0.5 ns) and late (ToF = 0.9 ns) times-of-flight, demonstrating an increase in cerebral blood flow during stimulation compared with control, consistent with a localized cortical response. Shaded areas indicate standard error.

Figure 9 presents the group-averaged responses across all the subjects. Figures 9(a) and 9(b) show the relative changes in arterial blood pressure (rABP) and heart rate (rHR) for stimulation (solid red) and control (dashed red) conditions. Although no consistent or significant change in ABP across subjects (0.02±0.38%, p=0.96) was observed throughout the trial duration, HR showed a small transient decrease (−0.9±0.3%, p=0.02).

Figures 9(c)–9(f) illustrate the mean rBFi responses at early (0.2 and 0.3 ns), intermediate (0.5 ns), and late (0.9 ns) ToFs, respectively. At 0.2 ns [Fig. 9(c)], no discernible difference is found between stimulation and control conditions (1.3±0.8%, p=0.13), consistent with the extracerebral origin of this signal. By contrast, the ToF gate centered at 0.3 ns [Fig. 9(d)] demonstrated a small but statistically significant difference (2.6±0.9%, p=0.02) between stimulation and control trials, whereas the intermediate and late ToF gates at 0.5 ns [Fig. 9(e)] and 0.9 ns [Fig. 9(f)] reveal a pronounced increase in rBFi following stimulus onset (5.5±1.9%, p<0.05 and 12.3±2.6%, p<0.05, respectively). The functional hyperemic response, therefore, becomes more pronounced and results in higher-amplitude changes in flow at longer ToFs. The peak response occurs at 15 s post-stimulus onset, consistent with a hemodynamic response originating in the cerebral cortex. Control trials do not show any such increase at any ToF. Shaded areas in Fig. 9 represent standard error, indicating relatively low inter-subject variability, even at a ToF of 0.9 ns.

## 4 Discussion

This study introduces an advanced, parallelized time-of-flight-resolved interferometric neuromonitoring technology capable of measuring tissue blood flow at high acquisition rates and at late photon flight times. The CoMind R1 system incorporates significant innovation across all elements of its design and in the associated data processing pipelines.

The key to the performance of the device is a high-power, 1064 nm DFB laser that is driven with an iteratively optimized 200 kHz sawtooth sweep. This innovation, along with the use of multimode delivery and collection fibers to increase optical throughput, significantly improves device performance relative to prior implementations.

Motion of the multimode collection fiber relative to the single-mode fiber bundle could, in principle, alter the instantaneous speckle distribution at the coupling interface. However, such effects influence the detected interferometric signal only if the motion-induced speckle fluctuations occur on timescales comparable to the intrinsic speckle decorrelation of the sample. In biological tissue, decorrelation times are typically on the order of 1 to 100  μs. By contrast, mechanical drift or vibrations of the MMF coupling typically occur on millisecond-to-second timescales. We have not observed a heightened sensitivity to fiber motion associated with this fiber configuration.

CoMind R1 also overcomes the significant challenge of processing the very large volumes of data that result from time-resolved interferometric measurements. Using a custom ADC and GPU-enabled processing, the architecture sustains >25  Gb/s data throughput to provide pulsatile BFi measurements at any selected ToF in real time.

CoMind R1 continuously records interferometric data across a wide range of photon flight times, preserving the complete TPSF for each acquisition. For simplicity, our real-time processing currently performs BFi fitting at a single, user-selected ToF to provide immediate feedback for optimizing probe coupling and signal quality. However, in contrast to previously reported systems that undertake path length or coherence gating and acquire at one ToF at a time,^37^^,^^38^ CoMind R1 measures the entire ToF distribution simultaneously, with all TPSF and $G_{1}$ data stored for offline multi-ToF analysis. The FPGA-GPU framework employed in CoMind R1 is readily capable of supporting real-time multi-ToF fitting in future software releases. This framework is also suitable for large-scale manufacture using standard components that are widely available.

To assess the fundamental performance and temporal stability of the CoMind R1 system, we conducted continuous measurement of an intralipid-based phantom over a 24-h period. The associated TPSFs provided by CoMind R1 [Fig. 4(a)] exhibit a smooth leading edge and a single-exponential tail, free of detector after-pulsing artifacts or other confounds.

The IRF in swept-source interferometric systems is primarily determined by the sweep bandwidth and linearity of the laser sweep. A broader sweep bandwidth produces a narrower temporal IRF, improving theoretical time-of-flight resolution, but at the expense of reduced instantaneous optical power, because the output energy is distributed over a wider spectral range. Conversely, narrowing the sweep bandwidth increases the average optical power incident on the sample, improving photon statistics and SNR.

It is important to note that in choosing to pursue interferometric, time-of-flight-resolved methods, we deliberately selected a technology with an extremely broad range of potential clinical applications once fully exploited. With CoMind R1, we intentionally chose to operate with a reduced sweep bandwidth to maximize delivered optical power and therefore deep-layer sensitivity—an appropriate trade-off given the stated goal of CoMind R1 is to maximize our sensitivity to CBF. As shown in Fig. 4(a), the chosen sweep bandwidth yielded an IRF FWHM of 120 ps and a ToF-axis sampling resolution of 66 ps.

For comparison, earlier iNIRS instruments that achieved narrower IRFs (e.g., 35 ps in Kholiqov et al.^41^) used wider spectral sweeps but at significantly lower per-channel optical power. The IRF width of CoMind R1 is still appreciably narrower than values reported for most published TD-DCS systems^24^^,^^26^^,^^51^ and is also narrower than that of the best-in-class commercial TD-NIRS devices.^52^

As shown in Fig. 4, the system exhibits excellent temporal stability, with the standard deviation of the measured decay rates at a 50 Hz sampling rate equal to ∼1 to 2 kHz up to and beyond a ToF of 1 ns. To assess how this metric of signal quality scales with $g_{1}$ decay rate, we performed a series of experiments on the homogeneous phantom where $g_{1}$ decay rate was gradually increased by modifying the reduced scattering coefficient of the intralipid solution. Figure 5(d) demonstrates that the standard deviation at 1 ns remains in the range of 1 to 2 kHz even for high decay rates beyond 100 kHz.

Using the conservative standard deviation derived from 24-h phantom stability measurements of 1.7 kHz at 1 ns ToF, and assuming the median *in vivo* diastolic to systolic range of decay rates (15 to 50 kHz), CoMind R1 exhibits a coefficient of variation (CoV) at 1 ns of 3.4% at systole, 11.3% at diastole, and 6.1% for the mean *in vivo* decay rate value of 28 kHz. In practice, these CoV values can be substantially and justifiably reduced through temporal averaging: e.g., reporting the average pulse waveform using a rolling window of ten consecutive pulses yields effective CoVs of 1.1% at systole and 3.6% at diastole. For clinical monitoring, where CBF trends could very reasonably be reported at ∼1  Hz, the effective CoV based on the mean *in vivo* decay rate is reduced to 0.86% at 1 ns ToF. Relatedly, based on the standard sample-size formula for estimating a mean under a normal approximation, to measure the mean *in vivo* decay rate value of 28 kHz at 1 ns with 95% confidence, one would need to average ∼20 samples.

The multilayer phantom results depicted in Fig. 6(b) demonstrate that the system is sensitive to physiologically reasonable flow rates in a layer at the average adult scalp-to-brain depth of 12.5 mm. The flow settings for the bi-layer phantom were chosen to represent realistic superficial and deep flow regimes rather than to impose an assumed fixed contrast ratio. This approach ensures that the recovered $g_{1}$ decay rates span a physiologically relevant range observed *in vivo* across all ToF, allowing quantitative assessment of depth-dependent flow sensitivity. Our process of calibrating our multilayer qualitative phantom (though approximate) resulted in an upper bound on deep-layer (“brain”) flow that was 3.3 times that of the superficial (“scalp”) layer. This value is consistent with but at the lower end of the expected brain-to-scalp flow ratio *in vivo* of 3 to 10.^53^^,^^54^ Changes in the gradient of the $g_{1}$ decay rate versus ToF curve begin to become evident at a ToF of ∼0.4  ns, which can be considered the earliest point at which we obtain measurable sensitivity to the deep layer. This finding aligns well with prior functional TD-DCS and interferometric NIRS research (and our own visual stimulation results) that show that with sufficient averaging, brain-specific functional blood flow changes can be resolved *in vivo* by leveraging photon gates around 0.4 to 0.6 ns.^25^^,^^55^

However, the results indicate that even with a measurement noise as low as that of CoMind R1, to resolve the physiologically reasonable variations in deep layer flow depicted in Fig. 6(b) requires access to later ToFs—likely at 1 ns or beyond. Figure 6(b) shows that CoMind R1 is able to resolve a spread of $g_{1}$ decay rates created by varying the deep layer flow, even when flow in the superficial layer is significant. However, these states are only distinguishable at sufficiently late ToF.

It is important to note that the measured field autocorrelation functions associated with our multilayer flow phantom will intrinsically represent a weighted mixture of both diffusive and flow-driven motion, whereas measures of tissue are better approximated by a purely diffusive model. Our goal was to simulate the range of autocorrelation decay rates observed in tissue across all ToF in a dynamically controllable fashion to qualitatively demonstrate the dynamic range and sensitivity of CoMind R1. This phantom represents a trade-off between model ideality and experimental controllability. Phantoms based on pump-driven flow are commonplace,^56^^–^^58^ but multilayer phantoms that provide purely diffusive scatterer motion can be constructed via other means, including through manipulation of phantom temperature and viscosity.^59^

As the core goal of the development of CoMind R1 is to capture the pulsatile dynamics of blood flow in the brain, the quality of the BFi pulse waveforms generated by our devices as a function of ToF is a critical performance characteristic. Figure 7 provides examples of both high and low pulsatile BFi signal quality captured within a cohort of N=25 healthy volunteers. Using a custom metric to quantitatively assess pulsatile signal quality, we are able to derive a “corner ToF” value that represents the longest ToF at which a high-quality pulsatile BFi can be extracted. The distribution of the Corner ToF over 25 subjects is shown in Fig. 8, demonstrating a median value of 1.2 ns. The worst subject exhibited a corner ToF of 0.9 ns. This demonstrates the capacity of CoMind R1 to consistently measure high-quality, pulsatile BFi time series at late times-of-flight beyond 1 ns. We consider this to be an important result; one that shows that CoMind R1 significantly exceeds the performance of the prior state-of-the-art. No interferometric NIRS system has yet demonstrated pulsatile BFi measurements beyond ∼400 to 500 ps.^33^^,^^40^^,^^41^

Several groups have recently demonstrated alternative interferometric methods that provide ToF-gated measurements of BFi.^37^^,^^38^ These systems achieve ToF gating by manipulating source coherence and pathlength differences between reference and sample arms. However, such methods typically employ relatively wide ToF gates to extract BFi at longer photon flight times. For example, Robinson et al.^38^ demonstrated a pulsatile BFi signal with a gate centered as late as 800 ps, but with a width of 500 ps. The incorporation of earlier times-of-flight within the gate will impact depth specificity particularly because exponentially larger numbers of photons will be collected at earlier points within the gate. This should be compared with the results presented here that demonstrate the ability to recover pulsatile BFi time series at 1.2 ns with an IRF width of 120 ps. It is also important to note that current time-gated interferometric systems typically acquire data from a single selected pathlength or time-of-flight at any one time. By contrast, CoMind R1 employs an architecture that enables simultaneous recording of BFi at times-of-flight across the full TPSF range at high speed.

How late in ToF a device needs to be able to measure to provide sufficient brain sensitivity remains a critical question. An extensive Monte Carlo simulation work undertaken by our team was recently presented^60^ and forms a companion paper submitted concurrently with this work. These simulations show that at ToFs exceeding ∼1  ns, the recovered pulsatile BFi is more representative of the brain than the scalp for a typical adult with a brain depth of 12.5 mm. A ToF of 1.2 ns (as achieved here) is sufficient to ensure that the recovered BFi is more representative of the brain than the scalp in 85% of the adult population. A ToF of 1.5 ns, which we have also achieved in multiple subjects (Fig. 8), is sufficient to meet this threshold in 95% of adults.

As an alternative to pursuing time-resolved measurements and gating at late ToF, increasing SDS can also be used to enhance brain sensitivity. Operating at a sufficiently long SDS is, in fact, critical to ensuring the efficacy of CW diffuse optics methods.^20^ Prior work using CW and multi-exposure iDWS has demonstrated pulsatile BFi signals at SDS as large as 40 mm^61^ or even 50 mm,^62^ which are expected to provide brain sensitivities comparable to those achieved in this work.^60^ Zhou et al.^61^ reported a result closely resembling our Fig. 7(a), in which pulsatile waveforms were presented for increasing SDS (rather than ToF) up to 40 mm. These CW approaches are promising, but demonstrating brain sensitivity does not, in isolation, ensure a sufficiently brain-specific signal for clinical translation. Additional steps are necessary to isolate a brain-specific BFi signal, and those steps are very likely to require an independent measurement of scalp blood flow to support signal de-mixing. Time-resolved measurements offer another advantage here: by capturing BFi at multiple ToFs, they provide a degree of depth discrimination that cannot be achieved in the CW domain without a multi-SDS configuration.

To demonstrate this point and further validate the system’s in-vivo performance, we conducted measurements on adult subjects during a visual stimulation paradigm. Figure 9 presents compelling evidence that CoMind R1 delivers functional specificity, depth specificity, and brain sensitivity; visual stimulation produced a distinct increase in BFi, but only at later ToF, with no detectable response at 0.2 ns. The heart rate showed a small transient decrease (of ∼1%) while ABP remained stable.

These results demonstrate the utility of ToF-resolved methods in separating BFi signals from different cerebral compartments. This is the first time to our knowledge that ToF-specific (and thus depth-specific) functional hyperemic responses have been demonstrated with time-resolved interferometric technique. When compared with the functional TD-DCS results presented by Ozana et al.,^25^ the presented results show enhanced separability of the superficial and cerebral layers.

Taken together, the results presented here demonstrate that CoMind R1 is capable of leveraging the advantages of time-resolved interferometric methods for the measurement of tissue blood flow and achieves the low measurement noise, high sampling rate, high ToF resolution, and late times-of-flight necessary to monitor pulsatile blood flow issues with high cerebral sensitivity and specificity in a robust, scalable, and cost-effective architecture.

As part of the ongoing development and productization of the CoMind R1 platform, several areas of future research are also envisioned. We are expanding our *in vivo* testing to include larger, more diverse cohorts, including in clinical contexts. We are currently undertaking an extensive multisite feasibility study of adult intensive care patients using the clinical equivalent of CoMind R1. We are also in the process of undertaking an extensive comparison of CoMind R1 against transcranial Doppler ultrasound; one of the few other methods of noninvasively assessing cerebral blood flow. Beyond these ongoing research studies, the ultimate validation of noninvasive CBF measurements will require comparison against established quantitative flow imaging modalities such as MRI-ASL and 15O-PET, which remain the clinical gold standards for cerebral perfusion. Over the coming months, we also intend to establish collaborations with other research teams at a range of institutions to support research activity using CoMind R1 in other patient groups.

Building on what we have learnt with CoMind R1, work has already begun to develop a multiwavelength, time-of-flight resolved interferometric measurement platform to provide not only measures of CBF with the robustness and sensitivity described here but also to provide measurements of absolute tissue optical properties and tissue oxygen saturation.

## 5 Conclusions

In this study, we have described and validated CoMind R1—a parallelized, time-resolved interferometric optical sensing platform for noninvasive monitoring of pulsatile cerebral blood flow. Through a combination of novel phantom experiments and *in vivo* measurements, we have demonstrated that CoMind R1 reliably acquires low-noise, pulsatile blood flow measurements at photon times-of-flight well in excess of 1 ns, exceeding the prior art. We also explicitly demonstrate that, at these late times-of-flight, CoMind R1 provides sensitivity to both the deep layer of an anatomically scaled multilayer phantom and to the adult visual cortex *in vivo*. Although significant work remains, we believe that CoMind R1 overcomes the primary challenges associated with the optical monitoring of human cerebral blood flow. It is therefore our expectation that this technology will accelerate the clinical translation and wider adoption of noninvasive optical neuromonitoring technologies.

## Supplementary Material

10.1117/1.NPh.13.2.025002.s01

## Data Availability

Data and code underlying the results presented in this paper are not publicly available at this time but may be obtained from the authors upon reasonable request.
