# Supplementary material for: CoMind R1: a time-resolved interferometric optical neuromonitoring system for pulsatile cerebral blood flow measurement at late times-of-flight
Source: Neurophotonics. 2026 Mar 30;13(2):025002. doi: 10.1117/1.NPh.13.2.025002 (PMC13033898; doi:10.1117/1.NPh.13.2.025002)
Supplement: Supplementary file 1 [file NPh_013_025002_SD001.pdf]

Supplementary Material

1. *Bland-Altman plot of g_1 decay rate with and without denoising*

Figure S1 displays Bland-Altman plots comparing g_1 decay rate for the at-rest in-vivo dataset of 25 subjects, processed both with (ξ_d) and without (ξ_0) denoising for two times-of-flight (0.2 ns and 1 ns). A window of 500 samples (~10s) per subject was taken for the analysis which is consistent with the denoising algorithm described in the Methods section. Comparison of decay rates fitted with and without denoising revealed statistically significant but negligible mean biases for both conditions (0.2 ns: 0.06 kHz, $p < 0.001$; 1 ns: -0.5 kHz, $p < 0.001$; Wilcoxon signed-rank test). The 0.2 ns condition showed excellent agreement with narrow limits of agreement (±0.5 kHz), indicating that denoising has minimal impact on the fitting when the decay rate is slow. However, the 1 ns condition exhibited wider limits of agreement (±5.6 kHz) with variability increasing with the mean decay rate.

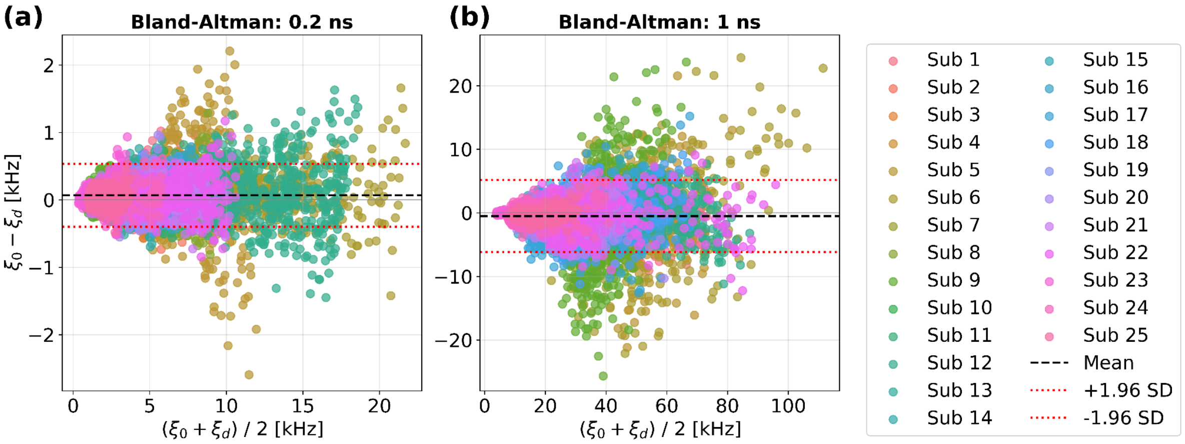

Fig. S1 Bland-Altman plots comparing g_1 decay rates for the at-rest in-vivo dataset with 25 subjects (500 consecutive data points per subject) processed both with (ξ_d) and without (ξ_0) denoising. The plots show example ToFs of 0.2 (a) and 1 ns (b).

2. Autocorrelation functions from multi-layer dynamic flow phantom.

Figure S2 presents autocorrelation functions and their respective fits from the multi-layer flow phantom experiment for the four most extreme cases (combinations of minimum and maximum flow in both layers). The fitting algorithm described in the manuscript was restricted to the upper portion of the autocorrelation function (as indicated by the dashed lines in Figure S2).

Autocorrelation functions in heterogeneous media can exhibit multi-exponential behavior, and such behaviour is expected for the cases where the flow rate in the two layers of this phantom is significantly different. However, as shown in Figure S2, a single-exponential model provides a reasonable characterization of the g_1s exhibited by this phantom when fitting is constrained to the early lags.

Since the design of the flow phantom would imply the presence of both diffusion and random flow induced by the pump, we also attempted to fit the data with an augmented model which includes both diffusion and random flow terms in the form:

$$g_1 = e^{-D\tau-\beta\tau^2}$$

where D is an effective diffusive-like decorrelation rate and β reflects a random flow contribution.[11,40,63] When fitting is restricted to early lags (the range above the dashed lines in Fig. S2), the augmented model is practically indistinguishable from the original single exponential implementation and the τ^2 term is negligibly small. When both models are applied to the full $g_1(\tau)$ (black and red curves in Fig. S2), the inclusion of the τ^2 term does not consistently improve the recovery of the shape of the experimental data and yields results very similar to the original single-exponential model. The residuals of the fit obtained with the augmented model were comparable to those from the single-exponential model across

ToF gates and flow conditions. This occurs even though an exponential function containing both τ and τ^2 terms would, in principle, be expected to fit the data more closely, as it represents a second-order approximation.

To investigate this behavior, we examined the covariance matrix returned by the fitting procedure. The resulting correlation coefficient between the fitted parameters D and β was consistently high ($|corr(D,\beta)| \approx 0.9$ across all flow rates and ToF gates), indicating strong coupling between the linear and quadratic decay terms. In this situation, changes in D can be compensated by corresponding changes in β while producing nearly identical autocorrelation curves, meaning the two parameters cannot be independently resolved from the measured data. As a result, the augmented model effectively reduces to a single effective decay rate, which is consistent with the observation that the τ^2 term does not lead to a systematic improvement in the fits.

It is important to note that neither model explicitly accounts for the multi-layered structure of the phantom and, therefore, they both suffer similar fitting discrepancies.

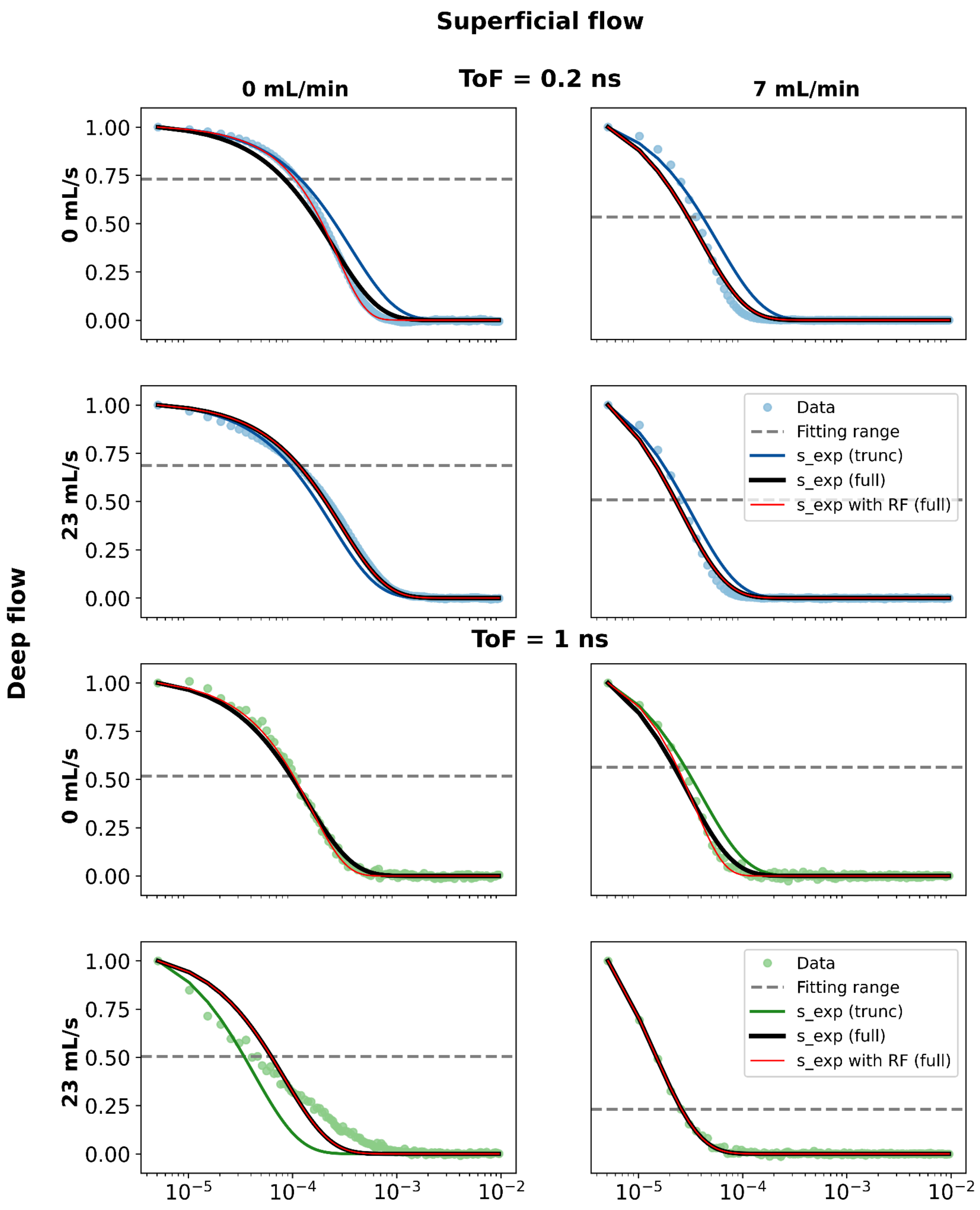

Fig. S2 A selection of the autocorrelation functions measured in the multi-layer flow phantom experiments: the top 4 subplots - at ToF = 0.2 ns with light blue points representing the measured autocorrelation function and the solid blue line corresponding to the single exponential model applied to truncated g_1 as described in Methods; the bottom 4 subplots - at ToF = 1 ns with light green points representing the measured autocorrelation function and the solid green line corresponding to the truncated single exponential fit. The horizontal gray dashed lines mark the lower edge of the truncated fitted range. Black and red curves correspond to the fits obtained with the single exponential model with and without random flow (RF) term respectively, as applied to the full range of lags.

3. Signal-to-noise ratio metric for assessing system signal quality

To assess device signal quality, a signal-to-noise ratio (SNR) metric can be calculated as follows:

$$SNR = 10log_{10}(\frac{TPSF_{ampl}}{TPSF_{noise}})$$

where $TPSF_{ampl}$ is the amplitude of the TPSF (extracted at 5 Hz) and is equivalent to the amplitude of the autocorrelation function, $TPSF_{noise}$ is a standard deviation over the ToF range [$TPSF_{peak}$ + 2 ns, $TPSF_{peak}$ + 3 ns]. This metric is calculated for the data acquired on the long-duration homogeneous liquid phantom described in the Methods section. However, the use of this metric for performance comparisons with other interferometric or time-domain systems must be undertaken with caution as this metric is strongly dependent on device acquisition settings including sweep range, sweep bandwidth, sweep rate, and ToF resolution. An example of the per-channel SNR calculated for the CoMind R1 system is shown in Figure S3.

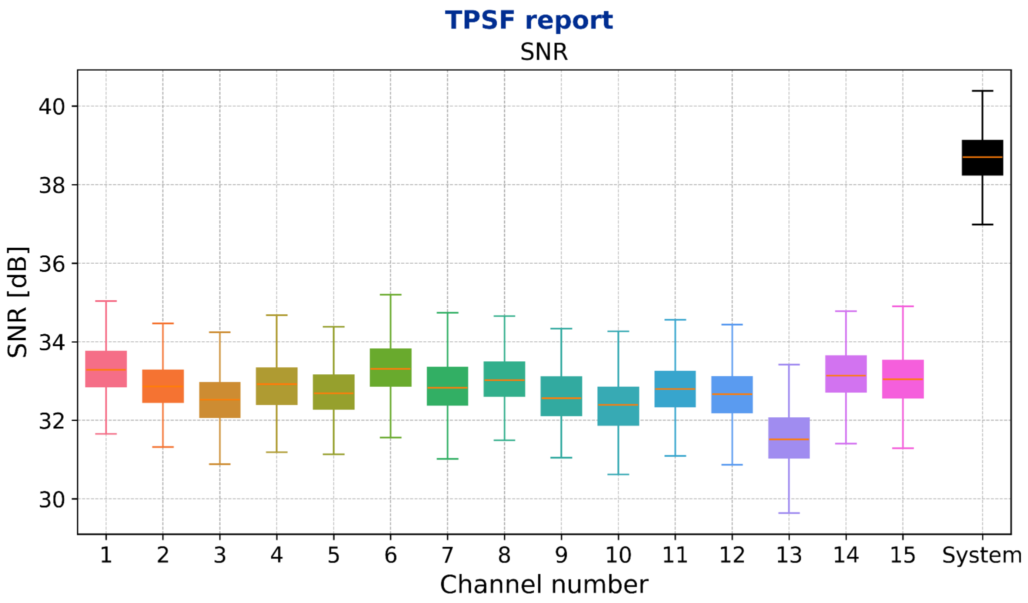

Fig. S3 An example of the per-channel SNR metric calculated on the data acquired over 2 minutes with the long-duration homogeneous phantom. The black boxplot shows SNR of the whole system with all the channels combined.
